# Novel inducible nitric oxide synthase-inhibiting cytochalasins from an oyster-derived fungus *Westerdykella dispersa* Ca4-13: structural insights and molecular docking analysis

**DOI:** 10.1186/s40529-025-00481-z

**Published:** 2025-10-07

**Authors:** Shu-Jung Huang, Su-Jung Hsu, Shih-Wei Wang, Yi-Chien Liu, Cheng-Yan Jiang, George Hsiao, Tzong-Huei Lee

**Affiliations:** 1 Institute of Fisheries Science, National Taiwan University, Taipei, 106319 Taiwan; 2 Department of Medicine, MacKay Medical College, New Taipei City, 25245 Taiwan; 3 Institute of Biomedical Sciences, MacKay Medical College, New Taipei City, 25245 Taiwan; 4 School of Pharmacy, College of Pharmacy, Kaohsiung, 807378 Taiwan; 5 Ph.D. Program in Drug Discovery and Development Industry, College of Pharmacy, Taipei Medical University, Taipei, 11031 Taiwan; 6 Department of Pharmacology, School of Medicine, College of Medicine, Taipei Medical University, Taipei, 11031 Taiwan; 7 Graduate Institute of Medical Sciences, College of Medicine, Taipei Medical University, Taipei, 11031 Taiwan; 8 Department of Life Science, College of Life Science, National Taiwan University, Taipei, 106319 Taiwan

**Keywords:** *Westerdykella dispersa*, Sporormiaceae, Westerchalasin, Anti‑inflammation, Molecular docking

## Abstract

**Background:**

Marine-derived microorganisms are renowned for producing structurally diverse secondary metabolites with notable biological activities, serving as a promising reservoir for pharmaceutical development. In this study, the fungal strain *Westerdykella dispersa* Ca4-13, isolated from the edible oyster *Crassostrea angulata*, was investigated for its potential anti-inflammatory and cytoprotective properties using BV-2 microglial cells as a model system.

**Results:**

Metabolite profiling of the solid-state fermented products of *W. dispersa* Ca4-13 yielded seven compounds **1**–**7**. Their structures were elucidated using NMR and MS techniques, revealing three previously undescribed cytochalasins, namely westerchalasin A (**1**), westerchalasin B (**2**), and westerchalasin C (**3**), along with four known compounds **4**–**7**. Among these, westerchalasin B (**2**) and westerchalasin C (**3**) significantly exhibited nitric oxide (NO) production production in LPS-stimulated BV-2 microglial cells, with IC₅₀ values of 11.1 ± 0.4 and 9.9 ± 0.4 µM, respectively. Western blot analysis demonstrated that compounds **2** and **3** significantly downregulated inducible nitric oxide synthase (iNOS) expression at a concentration of 20 µM. Moreover, molecular docking analysis revealed that compound **3** exhibited a high binding affinity for iNOS synthase (ΔG = -18.8104 kcal/mol). The strong interaction was attributed to of hydrogen bonds between the catalytic residue Arg375 and the C-18 carbonyl group of the cycloundecene moiety, as well as Pi-alkyl interactions with Trp367, which contributed to enhanced stability of the complex.

**Conclusions:**

This study reported the isolation and structural elucidation of three novel cytochalasins **1**–**3** from *W. dispersa* Ca4-13. Notably, compounds **2** and **3** demonstrated anti-inflammatory activity by inhibiting NO production and iNOS expression in LPS-stimulated BV-2 microglial cells. Molecular docking analysis further confirmed strong interactions between compound **3** and key iNOS residues. Given the crucial role of neuroinflammation in neurodegenerative disorders, these findings suggested that compounds **2** and **3** may possess dual neuroprotective properties, warranting further exploration for therapeutic applications.

**Supplementary Information:**

The online version contains supplementary material available at 10.1186/s40529-025-00481-z.

## Background

Marine-derived fungi have garnered significant scientific interests due to their ability to produce structurally diverse secondary metabolites with remarkable bioactivities (Hoang et al. 2018). Over the past decade, an increasing number of studies have reported the biodiversity, chemodiversity, and pharmaceutical potential of marine fungi, highlighting their role as a promising source of novel bioactive compounds (Bao et al. 2018; Boufridi et al. 2018; Ancheeva et al. 2020; Lee et al. 2021; Chang et al. 2022; Chang et al. 2023; Li et al. 2023). Given the ecological and chemical complexity of marine habitats, these fungi have evolved unique metabolic pathways, making them an important target for natural product discovery (Wang et al. 2009; Brakhage et al. 2011; Hridoy et al. 2022). Research on fungal secondary metabolism has broad implications, spanning natural product chemistry, chemical ecology, and even marine biotechnology (Jung-Schroers et al. 2018; Lee et al. 2021; Li et al. 2023). Marine microorganisms play a crucial role in the health and physiological functions of marine invertebrates, often forming symbiotic relationships that contribute to host homeostasis (Scanes et al. 2021).

Oysters (*Crassostrea* spp.), as filter-feeding bivalves with both ecological and economic significance, host a diverse array of microorganisms, yet the functional roles and bioactive potential of many of these microbes remain largely unexplored (Apprill 2017). Expanding research into the diversity, metabolic capacity, and bioactivities of oyster-associated fungi is essential to fully integrate fungi into the broader understanding of marine microbiology and biotechnology (Cunliffe 2023).

The fungal genus *Westerdykella*, first described by Stolk in 1955 (Stolk 1955), occur worldwide in a variety of substrates, including soil, mud, and plant materials (Ebead et al. 2012). Notably, *Westerdykella* species have been reported to produce cytochalasan-type secondary metabolites (Lin et al. 2009), which are structurally unique fungal metabolites characterized by a bicyclic lactam (isoindolone) core. To date, approximately 400 cytochalasans have been identified, exhibiting diverse biological properties such as antiviral, antibacterial, anti-inflammatory, and antitumor activities, as well as inhibiting cholesterol synthesis (Lin et al. 2009; Scherlach et al. 2010; Xu et al. 2017; Sallam et al. 2021).

Chronic inflammation is increasingly recognized as a key contributor to pathological conditions, including neurodegenerative diseases, where persistent immune activation leads to oxidative stress and neuronal damage (Jomova et al. 2023). The overproduction of reactive oxygen species (ROS) disrupts the balance between antioxidant defenses and oxidative stress, promoting DNA damage, epigenetic modifications, and aberrant signaling cascades (Kwak et al. 2020; Mittal et al. 2020; Yadid et al. 2020). In particular, ROS-mediated activation of the NF-κB signaling pathway leads to the upregulation of pro-inflammatory genes, exacerbating neuroinflammation and contributing to the pathogenesis of neurodegenerative disorders (Xu et al. 2022). Among the key inflammatory mediators, iNOS is a crucial enzyme responsible for excessive NO production, which plays a central role in inflammation-induced neuronal injury (Gupta et al. 2024). Consequently, targeting iNOS has emerged as a promising strategy for mitigating neuroinflammation and its associated pathologies.

In this study, the fungal strain *W. dispersa* Ca4-13 was isolated from the edible oyster *Crassostrea angulata* Lamarck (Crassostreinae), collected from Yunlin County, Taiwan. Preliminary biological evaluations revealed that crude extracts from *W. dispersa* Ca4-13 solid-state fermented products exhibited significant anti-inflammatory activity at a concentration of 100 µg/mL. This prompted further investigation, leading to the isolation and identification of seven constituents, including three previously undescribed cytochalasins. Herein, we report the anti-inflammatory potential of these compounds was assessed by evaluating their inhibitory effects on NO production in LPS-stimulated BV-2 microglial cells. Furthermore, their ability to suppress iNOS expression was examined using Western blot analysis. Additionally, molecular docking studies were performed to provide mechanistic insights into their interactions with iNOS. The findings highlight the potential of these novel iNOS-inhibiting cytochalasins as lead compounds for the development of neuroprotective agents.

## Results and discussions

### Identification of compounds isolated from the EtOAc extract of solid-state fermented products of W. dispersa Ca4-13

**Fig. 1:**
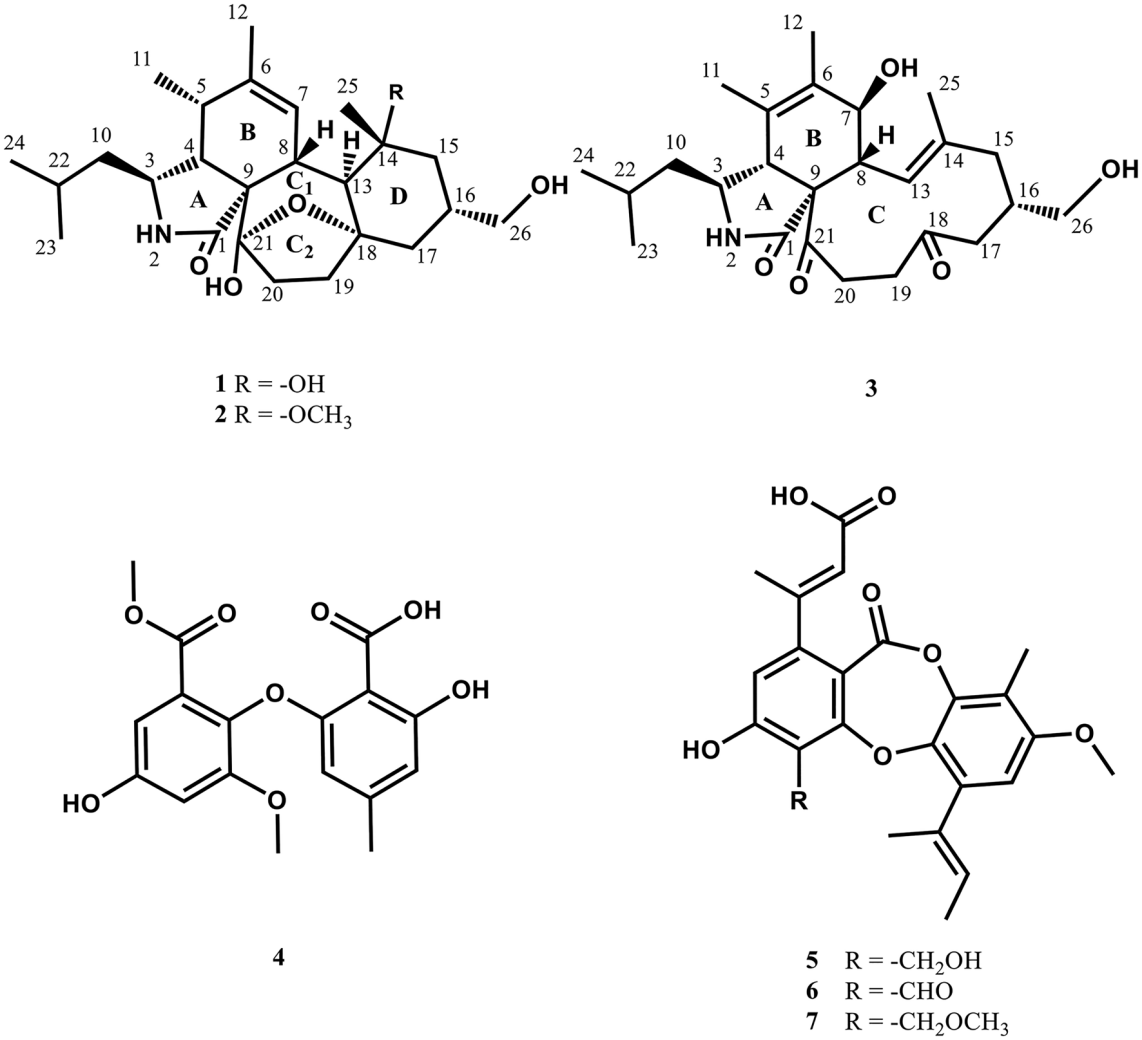
Chemical structures of compounds **1**–**7** identified in this study

The EtOAc extract of solid-state fermented products of *W. dispersa* Ca4-13 was fractionated and purified sequentially by column chromatography on Sephadex LH-20 and semipreparative HPLC to afford three previously undescribed compounds **1**–**3**, along with asterric acid (**4**) (Wang et al. 2016), auranticin A (**5**) (Poch et al. 1991), auranticin B (**6**) (Poch et al. 1991), and pilobolusone C (**7**) (Rajachan et al. 2014) (Fig. 1).

**Fig. 2:**
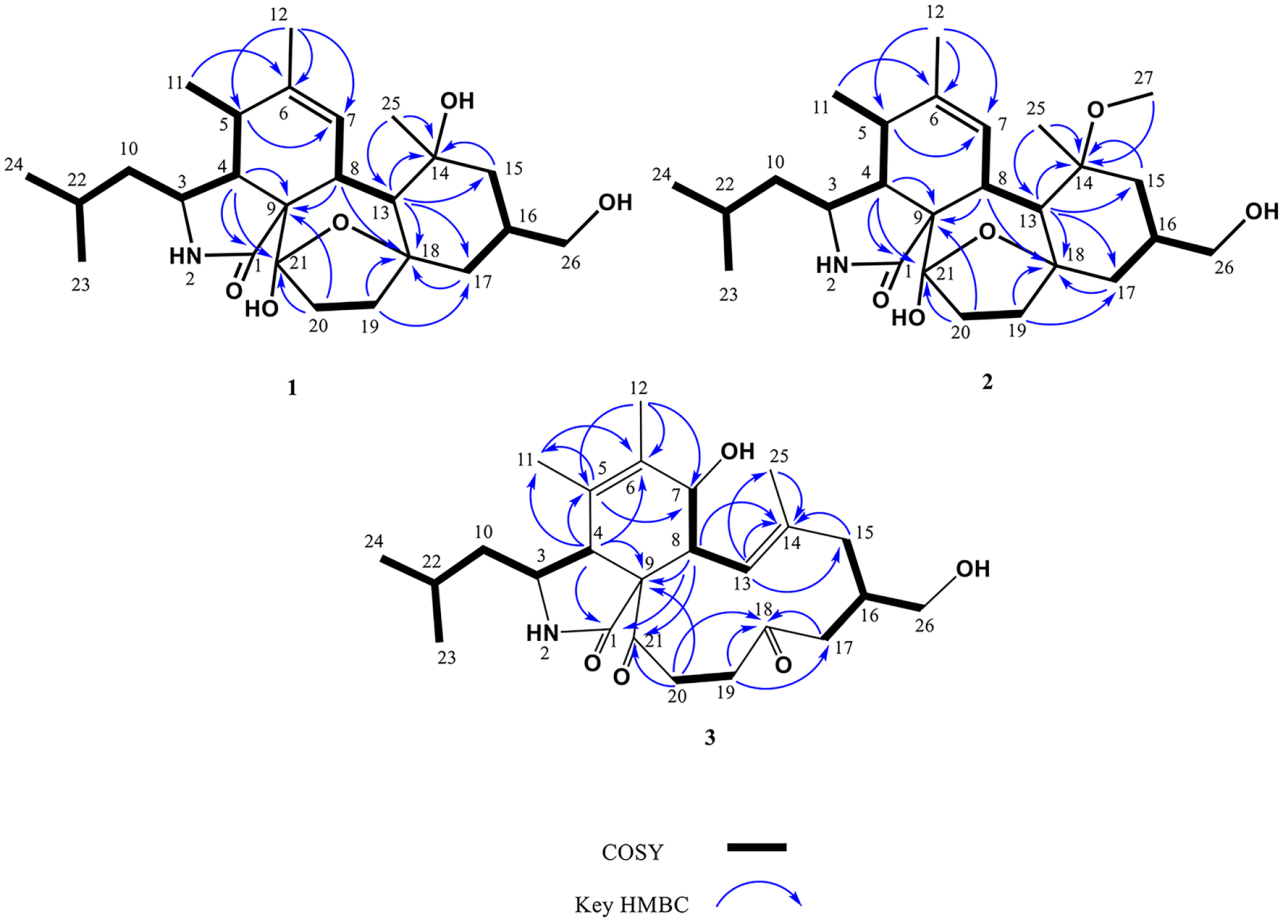
Key HMBC and COSY correlations of compounds **1**–**3**

**Fig. 3:**
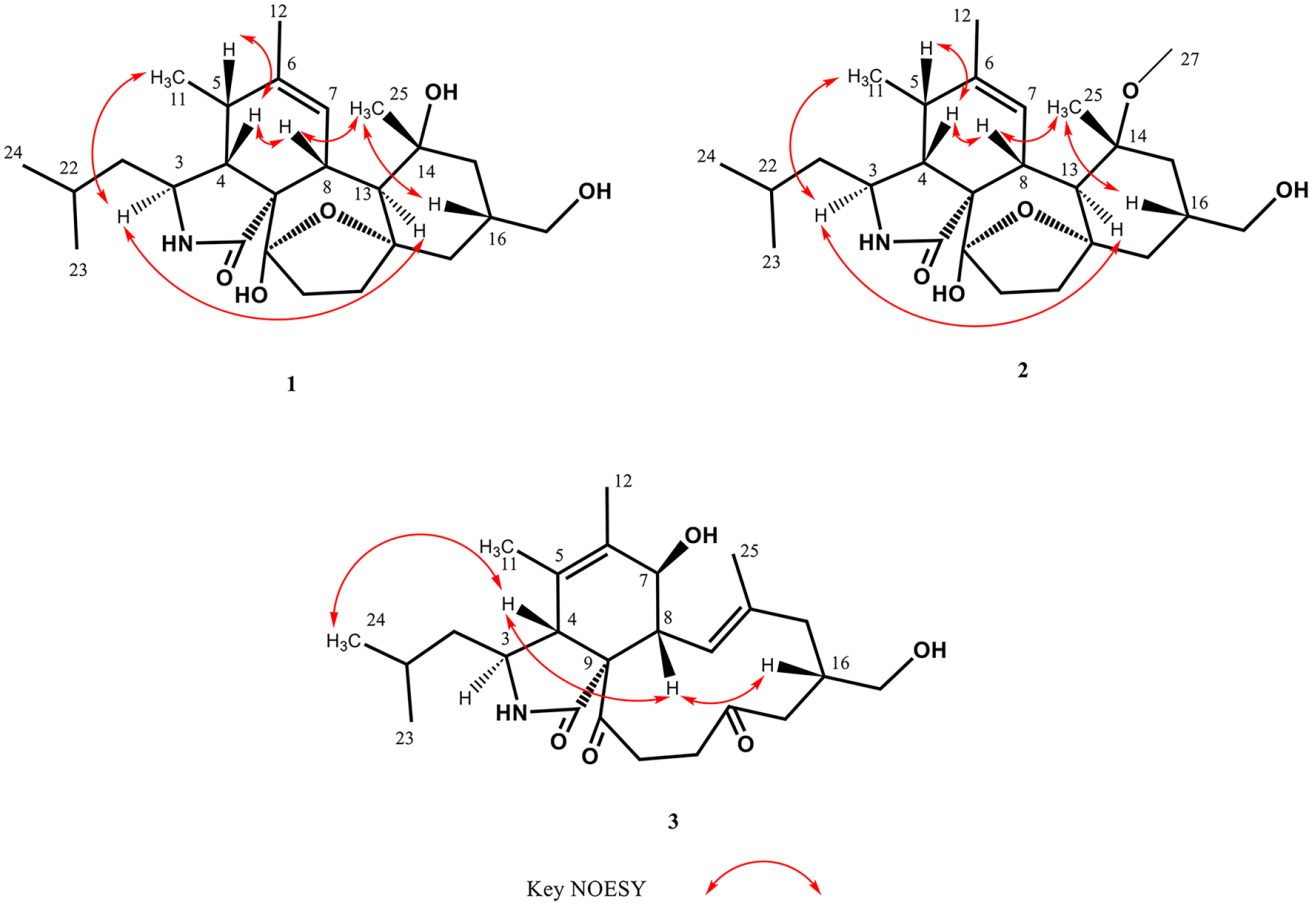
Key NOESY correlations of compounds **1**–**3**

**Fig. 4:**
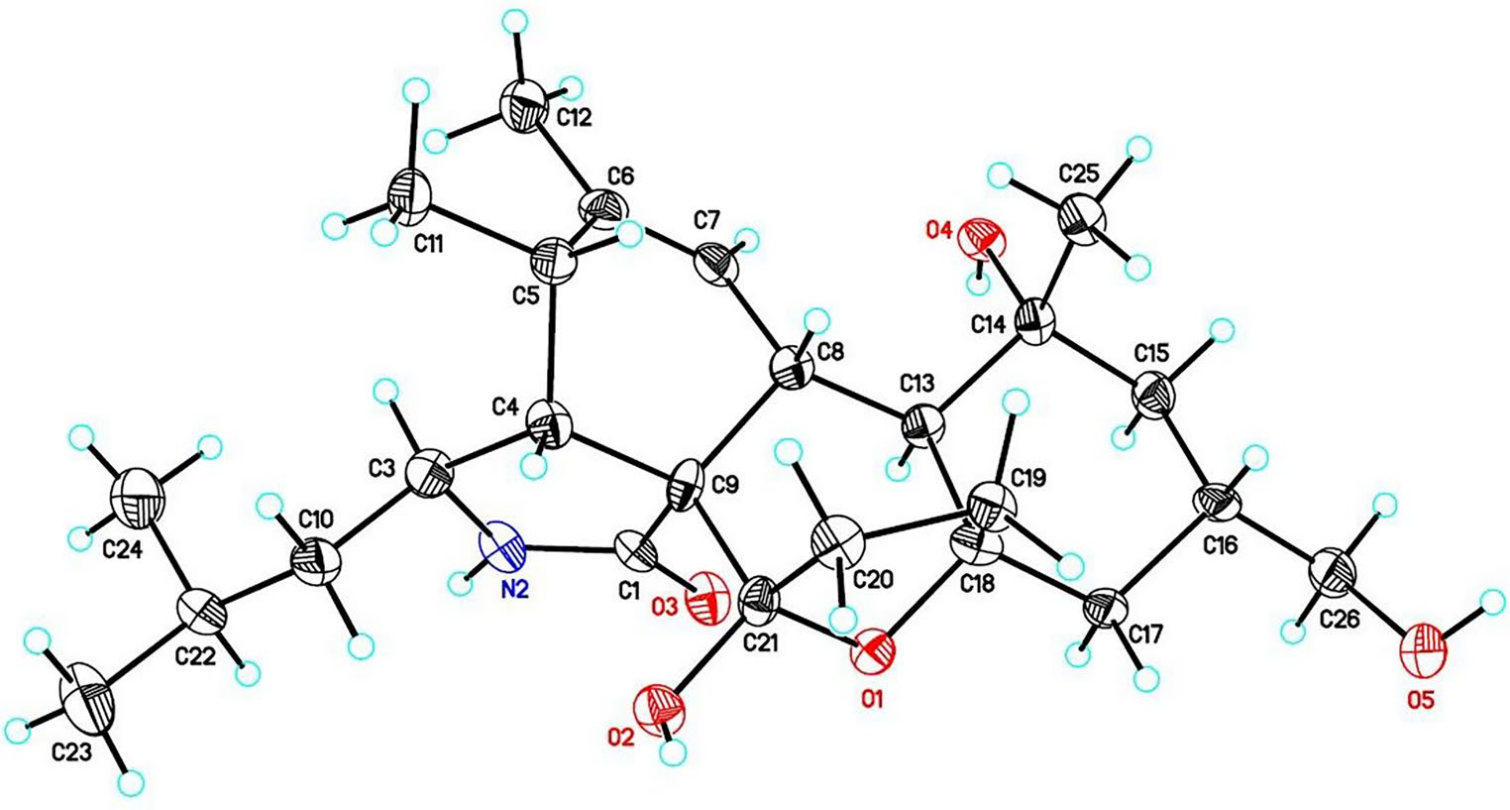
Computer-generated perspective drawings of the X-ray crystallographic model of compound **1**

Compound **1** was obtained as colorless crystal, and was determined to have the molecular formula C_25_H_39_NO_5_ as deduced from a protonated molecular ion [M + H]^+^ at *m*/*z* 434.2907 (calcd 434.2901 for C_25_H_40_NO_5_) in the HRESIMS (Fig. S1). Its IR spectrum revealed the presence of hydroxy (3347 cm^− 1^) and amide carbonyl (1647 cm^− 1^) functionalities (Fig. S2). Analysis of ^1^H NMR and HSQC spectra of **1** indicated signals for five methyls at *δ*_H_ 0.91 (3 H, d, *J* = 7.0 Hz, H-24), 0.92 (3 H, d, *J* = 6.9 Hz, H-23), 1.08 (3 H, s, H-25), 1.23 (3 H, d, *J* = 7.2 Hz, H-11), and 1.75 (3 H, s, H-12), and one olefinic proton at *δ*_H_ 6.28 (1H, brs, H-7), one oxygenated methylene at *δ*_H_ 3.39 (1H, d, *J* = 6.0 Hz, H_a_-26) and 3.40 (1H, d, *J* = 6.0 Hz, H_b_-26), five methylenes at *δ*_H_ 1.20 (1H, m, H_a_-10) and 1.73 (1H, m, H_b_-10); 1.28 (1H, m, H_a_-15) and 1.78 (1H, m, H_b_-15); 1.58 (1H, m, H_a_-17) and 1.76 (1H, m, H_b_-17); 1.61 (1H, m, H_a_-19) and 2.13 (1H, m, H_b_-19); 1.77 (1H, m, H_a_-20) and 2.18 (1H, m, H_b_-20), and seven methines at *δ*_H_ 1.49 (1H, m, H-16), 1.63 (1H, m, H-22), 2.08 (1H, dd, *J* = 2.8, 5.5 Hz, H-4), 2.29(1H, br d, *J* = 11.7 Hz, H-8), 2.52 (1H, dq, *J* = 5.5, 7.2 Hz, H-5), 2.97 (1H, d, *J* = 11.7 Hz, H-13), and 3.15 (1H, dt, *J* = 2.8, 9.7 Hz, H-3) (Table 1). Interpretation of the ^13^C NMR accompanied by HSQC spectra revealed 25 carbon signals attributable to five methyls at *δ*_C_ 13.9 (C-11), 20.6 (C-12), 22.1 (C-23), 22.8 (C-25), and 24.3 (C-24), and seven methines at *δ*_C_ 26.0 (C-22), 36.0 (C-16), 36.7 (C-5), 39.3 (C-8), 50.5 (C-13), 51.8 (C-4), and 52.6 (C-3), and an olefinic methine at *δ*_C_ 127.8 (C-7), and six methylenes at *δ*_C_ 33.5 (C-19), 35.6 (C-20), 42.6 (C-17), 48.0 (C-15), 48.4 (C-10), and 68.1 (C-26), and five nonprotonated carbons at *δ*_C_ 60.7 (C-9), 74.4 (C-14), 84.3 (C-18), 107.2 (C-21), 138.5 (C-6), and 179.4 (C-1). Cross-peaks of H-4/H-3 in the COSY spectrum along with cross-peaks of H-4/C-1 and H-4/C-9 in the HMBC spectrum (Fig. 2), supported the elucidation of the γ-lactam part (A ring) in **1**. Correlations of H-4/H-5 and H-7/H-8 in the COSY spectrum, together with cross-peaks of H-4/C-9, H-5/C-7, H_3_-11/C-6, H_3_-12/C-5, H_3_-12/C-6, and H_3_-12/C-7 in the HMBC spectrum (Fig. 2), confirmed the existence of the cyclohexene moiety (B ring). Cross-peaks of H-8/H-13 and H-19/H-20 in the COSY spectrum, accompanied with cross-peaks of H-8/C-9, H-8/C-18, H-19/C-18, H-20/C-21, and H-20/C-9 in the HMBC spectrum (Fig. 2), supported the presence of the cycloheptane part (C ring). Correlations of H_2_-15/H-16 and H-16/H_2_-17 in the COSY spectrum, together with cross-peaks of H-13/C-14, H-13/C-15, H-13/C-17, H-13/C-18, H-15/C-14, H_3_-25/C-13, and H_3_-25/C-14 in the HMBC spectrum (Fig. 2), corroborated the elucidation of the cyclohexane moiety (D ring). Based on HRESIMS analysis, compound **1** had seven degrees of unsaturation including an amide carbonyl (*δ*_C_ 179.4) and one double bonds (*δ*_C_ 127.8 and 138.5), indicating an additional pentacyclic ring system. A C-18 − O−C-21 oxygen bridge was thus proposed to satisfy the degrees of unsaturation and downfield shifts of C-18 (*δ*_C_ 84.3) and C-21 (*δ*_C_ 107.2). Moreover, the carbon chemical shift of C-21 at δ_C_ 107.2 revealed the presence of an additional hydroxy group at this position, implying a hemiketal group for C-21. Further assignments of all the correlations in the COSY as well as HMBC spectra (Fig. 2), the gross structure of **1** was thus established. In the NOESY spectrum of compound **1**, the cross-peaks of H-3/H_3_-11 and H-3/H-13 confirmed that H-3, H_3_-11, and H-13 were on the same side of the molecule (Fig. 3). The correlations of H-4/H-5, H-4/H-8, H-8/H_3_-25, and H_3_-25/H-16 confirmed that H-4, H-5, H-8, H-16, and H_3_-25 were on the same side of the molecule (Fig. 3). The large coupling constant (*J* = 11.7 Hz) between H-8 and H-13 suggested that H-8 and H-13 were located on opposite sides of the molecule. Above data confirmed that compound **1** shared the same relative configurations as those of flavipesine A (Zhang et al. 2019) to be 3S*,4R*,5S*, 8R*,13S*,14R*,16S*. For determining the absolute configurations of compound **1**, a single-crystal X-ray diffraction experiment with Cu *Kα* radiation (λ = 0.154 nm) was employed (Fig. 4). The absolute configurations of compound **1** were determined to be 3*S*,4*R*,5*S*,8*R*,9*S*,13*S*,14*R*,16*S*,18*S*,21*S*. Hence, compound **1** was characterized as shown in Fig. 1, and it was named westerchalasin A.

**Table 1:** ^1^H and ^13^C nuclear magnetic resonance data of compounds **1**–**3** (δ in ppm, mult., *J* in Hz)

	1 ^a^		2 ^a^		3 ^a^	
No.	^13^C	^1^H	^13^C	^1^H	^13^C	^1^H
1	179.4		179.4		177.2	
3	52.6	3.15 dt (2.8, 9.7)	52.6	3.14 dt (3.0, 10.1)	56.5	3.31 (overlap)
4	51.8	2.08 dd (2.8, 5.5)	51.8	2.08 dd (3.0, 5.5)	53.0	3.00 br
5	36.7	2.52 dq (5.5, 7.2)	36.7	2.50 dq (5.5, 7.2)	128.2	
6	138.5		138.9		134.8	
7	127.8	6.28 (br)	127.2	6.06 br	71.2	3.88 d (9.4)
8	39.3	2.29 (br, 11.7)	39.3	2.28 br	48.1	2.48 dd (9.4, 11.0)
9	60.7		60.6		63.6	
10a	48.4	1.20 m (overlap)	48.4	1.20 m (overlap)	46.8	1.13 m (overlap)
10b		1.73 m (overlap)		1.73 m (overlap)		1.16 m (overlap)
11	13.9	1.23 d (7.2)	13.9	1.23 d (7.2)	18.0	1.73 s
12	20.6	1.75 s	20.7	1.75 s	15.0	1.71 s
13	50.5	2.97 d (11.7)	45.1	3.30 (overlap)	125.9	6.08 d (11.0)
14	74.4		79.3		140.3	
15a	48.0	1.28 m (overlap)	40.5	1.45 m (overlap)	46.1	1.78 d (13.0)
15b		1.78 (m, overlap)		1.67 m (overlap)		2.23 d (13.0)
16	36.0	1.49 m	35.7	1.50 m (overlap)	35.9	2.54 m (overlap)
17a	42.6	1.58 m (overlap)	42.5	1.54 (m, overlap)	43.0	2.17 d (16.0)
17b		1.76 m (overlap)		1.72 m (overlap)		2.55 m (overlap)
18	84.3		84.4		210.7	
19a	33.5	1.61 m (overlap)	34.0	1.65 m (overlap)	38.8	2.63 (overlap)
19b		2.13 m (overlap)		2.17 m (overlap)		2.73 (overlap)
20a	35.6	1.77 m (overlap)	35.6	1.77 m (overlap)	38.9	2.58 m (overlap)
20b		2.18 m (overlap)		2.17 m (overlap)		3.79 brt (13.6)
21	107.2		107.2		209.0	
22	26.0	1.63 m (overlap)	26.0	1.63 m (overlap)	26.3	1.61 m
23	22.1	0.92 d (7.0)	22.1	0.92 d (6.2)	22.7	0.90 d (6.6)
24	24.3	0.91 d (7.0)	24.3	0.91 d (6.2)	23.4	0.93 d (6.6)
25	22.8	1.08 s	21.1	1.09 s	15.8	1.34 s
26a	68.1	3.39 d (6.0)	68.1	3.42 d (5.4)	67.8	3.36 m (overlap)
26b		3.40 d (6.0)		3.42 d (5.4)		3.47 m (overlap)
27			48.0	3.21 s		

^*a*^ Data were measured in CD_3_OD at 500 MHz for ^1^H and 125 MHz for ^13^C

**Fig. 5:**
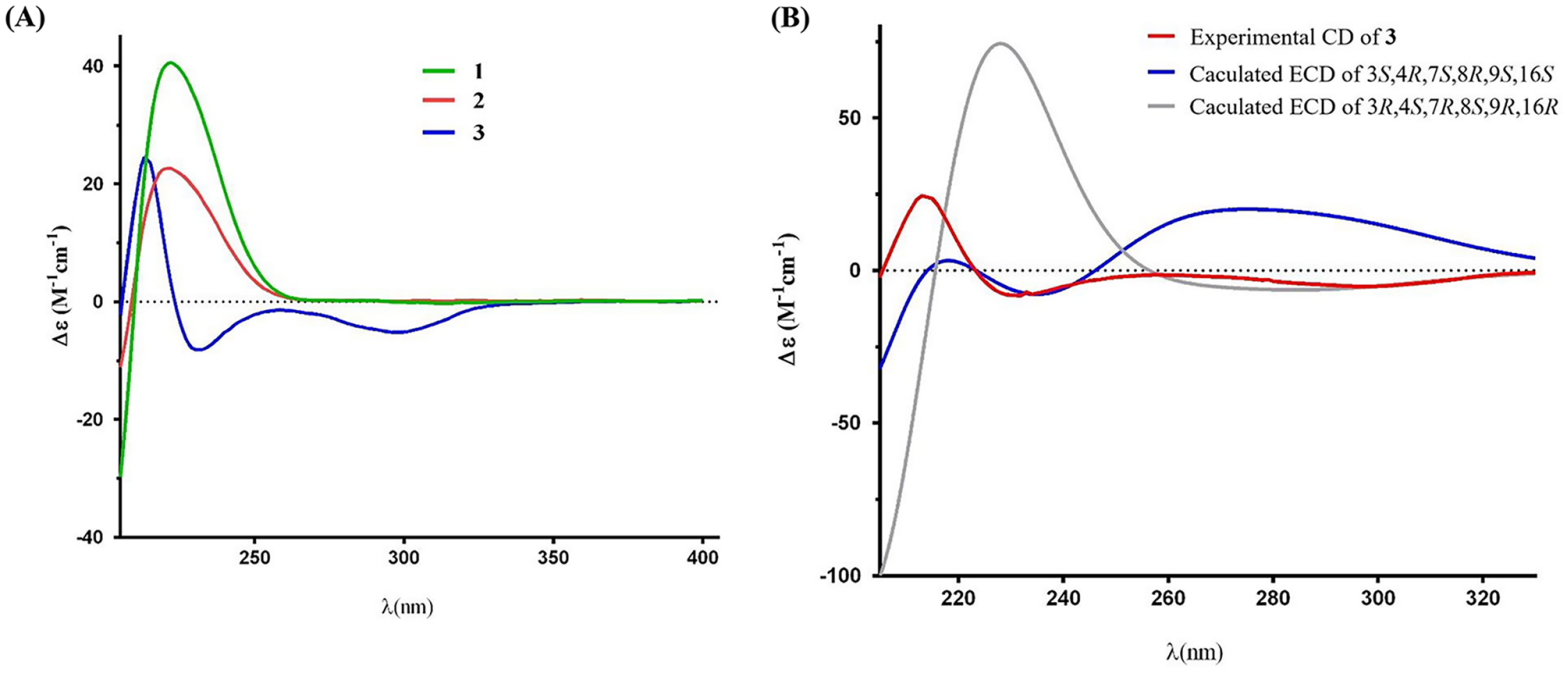
Experimental ECD spectra of compounds **1**–**3** (**A**) and calculated ECD spectra of compound **3** (**B**)

Compound **2** was obtained as whitish powder, and was determined to have the molecular formula C_26_H_41_NO_5_ as deduced from a protonated molecular ion [M + H]^+^ at *m*/*z* 448.3059 (calcd 448.3052 for C_26_H_42_NO_5_) in the HRESIMS (Fig. S9). Its IR spectrum revealed the presence of hydroxy (3343 cm^− 1^) and amide carbonyl (1666 cm^− 1^) functionalities (Fig. S10). The ^1^H and ^13^C NMR data of **2** revealed close structural similarities with those of **1** (Table 1), except for the presence of one additional methoxy at *δ*_H_ 3.21/*δ*_C_ 48.0. Based on a key HMBC correlation of H_3_-27 to C-14 (Fig. 2), the planar structure of **2** was established. In the NOESY spectrum of **2** (Fig. 3), the cross peaks of H-3/H_3_-11, H-5/H-8, H-3/H-13, H-8/H_3_-25, and H-16/H_3_-25 corroborated that **2** adopted the same relative configuration as that of **1**. The absolute configuration of **2** was deduced to have the same absolute configuration by comparing the experimental ECD spectrum of **2** with those of **1**, which showed the same positive Cotton effect at around 220 nm (Fig. 5). Thus, the absolute configuration of compound **2** was determined to be 3*S*,4*R*,5*S*,8*R*,9*S*,13*S*,14*R*,16*S*,18*S*,21*S*. Hence, compound **2** was characterized as shown in Fig. 1, and was named westerchalasin B.

Compound **3** was obtained as whitish powder, and was determined to have the molecular formula C_25_H_37_NO_5_ as deduced from a protonated molecular ion [M + H]^+^ at *m*/*z* 432.2746 (calcd 432.2744 for C_25_H_40_NO_5_) in the HRESIMS (Fig. S17). Its IR spectrum revealed the presence of hydroxy (3344 cm^− 1^) and carbonyl (1689 cm^− 1^) functionalities (Fig. S18). The ^1^H NMR (Table 1) and HSQC (Fig. S21) data of **3** indicated signals of five methyls at *δ*_H_ 0.90 (3 H, d, *J* = 6.6 Hz, H-23), 0.93 (3 H, d, *J* = 6.6 Hz, H-24), 1.34 (3 H, s, H-25), 1.71(3 H, s, H-12), and 1.73 (3 H, s, H-11), one olefinic proton at *δ*_H_ 6.08 (1H, d, *J* = 11.0 Hz, H-13), one oxygenated methylene at *δ*_H_ 3.36 (1H, m, H_a_-26) and 3.47 (1H, m, H_b_-26), five methylenes at *δ*_H_ 1.13 (1H, m, H_a_-10) and 1.16 (1H, m, H_b_-10); 1.78 (1H, d, *J* = 13.0, H_a_-15) and 2.23 (1H, d, *J* = 13.0, H_b_-15); 2.17 (1H, d, *J* = 16.0, H_a_-17) and 2.55 (1H, m, H_b_-17); 2.58 (1H, m, H_a_-20) and 3.79 (1H, br t, *J* = 13.6, H_b_-20); and 2.63 (1H, m, H_a_-19) and 2.73 (1H, m, H_b_-19), and six methines at *δ*_H_ 1.61 (1H, m, H-22), 2.48 (1H, dd, *J* = 9.4, 11.0 Hz, H-8), 2.54 (1H, m, H-16), 3.00 (1H, br s, H-4), 3.31 (1H, m, H-3), and 3.88 (1H, d, *J* = 9.4, H-7) (Table 1). Interpretation of the ^13^C NMR accompanied by HSQC spectrum revealed 25 carbon signals attributable to three carbonyls at *δ*_C_ 177.2 (C-1), 209.0 (C-21), and 210.7 (C-18), and four nonprotonated carbons at *δ*_C_ 63.6 (C-9), 128.2 (C-5), 138.5 (C-6), and 140.3 (C-14), one olefinic methine at *δ*_C_ 125.9 (C-13), six methines at *δ*_C_ 26.3 (C-22), 35.9 (C-16), 48.1 (C-8), 53.0 (C-4), 56.5 (C-3) and 71.2 (C-7), and six methylenes at *δ*_C_ 38.8 (C-19), 38.9 (C-20), 43.0 (C-17), 46.1 (C-15), 46.8 (C-10) and 67.8 (C-26), and five methyls at *δ*_C_ 15.0 (C-12), 15.8 (C-25), 18.0 (C-11), 22.7 (C-23), and 23.4 (C-24). Correlations of H-4/H-3 in the COSY spectrum along with cross-peaks of H-4/C-1 and H-4/C-9 in the HMBC spectrum (Fig. 2), confirmed the elucidation of the γ-lactam part (A ring). Cross-peaks of H-7/H-8 in the COSY spectrum together with correlations of H-4/C-5, H-4/C-6, H-4/C-9, and H-5/C-7 in the HMBC spectrum (Fig. 2), supported the existence of the cyclohexane moiety (B ring). Correlations of H-8/H-13, H-15/H-16, H-16/H-17, H-16/H-26, and H-19/H-20 in the COSY spectrum accompanied with cross-peaks of H-8/C-9, H-8/C-14, H-8/C-21, H-13/C-14, H-13/C-15, H-13/C-25, H-15/C-14, H-17/C-18, H-19/C-18, H-20/C-9, H-20/C-18, and H-20/C-21 in the HMBC spectrum (Fig. 2), established the 11-membered diketone-containing C ring. Further full assignments of all the correlations in the COSY and HMBC spectra (Fig. 2), the plain structure of **3** was assigned. In the NOESY spectrum, correlations of H-4/H-8 and H-8/H-16 indicated that H-4, H-8, and H-16 were on the same side, while the correlation between H-4 and H-24 and the absence of a cross-peak between H-3 and H-4 confirmed that H-3 and H-4 were positioned on opposite sides (Fig. 3). Furthermore, the large coupling constant (*J* = 9.4 Hz) between H-7 and H-8 suggested that H-7 and H-8 were also located on the opposite sides of the molecule. According to the literature, a significant positive Cotton effect in the 210–254 nm region of the ECD spectrum was observed when the isoindolone core of cytochalasin derivatives adopted an *R* configuration at C**-**4, and it was oriented in the same direction as C-9 (Zhang et al. 2019; Wang et al. 2025). The calculated ECD spectrum for 3*S*,4*R*,7*S*,8*R*,9*S*,16*S* configurations of **3** was consistent with the experimental ECD curve (Fig. 5), indicating compounds **3** and **1** had the same absolute configuration in main structural feature. Thus, the absolute configuration of compound **3** was determined to be 3*S*,4*R*,7*S*,8*R*,9*S*,16*S*. Hence, compound **3** was characterized as shown in Fig. 1, and it was named westerchalasin C.

### Effects of compounds 1–7 on cell viability and NO production in LPS-stimulated BV-2 microglial cells

**Fig. 6:**
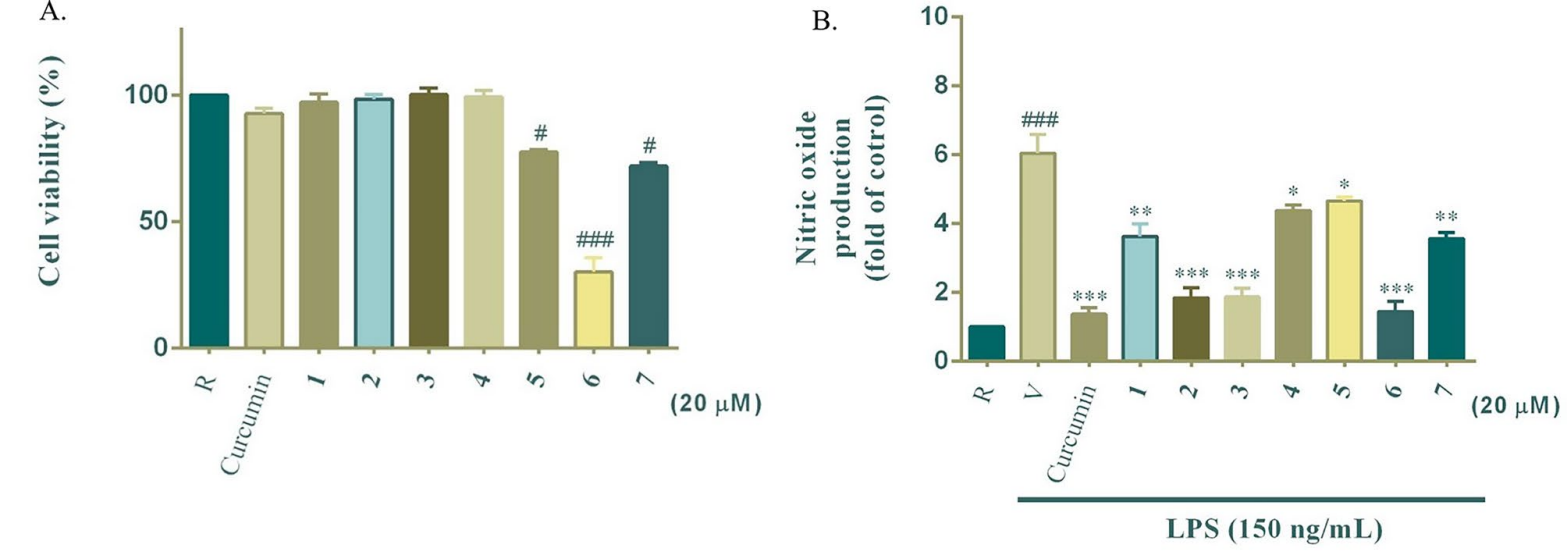
Cytotoxicity of compounds **1–7** in BV-2 microglial cells (**A**) and their effects on LPS-induced NO production (**B**). The concentration of test compounds was 20 µM. Data are expressed as the mean ± SD (*n* = 3). ^#^*p* < 0.05 and ^###^*p* < 0.001, compared with the resting group (R); ^*^p < 0.05, ^**^p < 0.01 and ^***^p < 0.001, compared with the group of stimulation (V)

To assess the potential cytotoxic effects of compounds **1**–**7**, the MTT assay was performed prior to evaluating their anti-neuroinflammatory activity. As shown in Fig. 6A, most of tested compounds exhibited low cytotoxicity at 20 µM, confirming their safety for subsequent NO inhibition assays. However, compound **6** notably reduced BV-2 cell viability to below 50%, suggesting potential cytotoxicity at the tested concentration. This result highlights the importance of evaluating both bioactivity and cytotoxicity when considering potential therapeutic applications. Moreover, among known compounds, asterric acid (compound **4**) analogs have been shown in previous studies to exhibit endothelin-binding inhibitory activity (Tan et al. 2024). Pilobolusone C (compound **7**) exhibited cytotoxicity against the MCF-7 cancer cell line (Rajachan et al. 2014) and mild cytotoxicity toward BV-2 microglial cells.

After confirming the cytotoxicity profiles, we evaluated the effects of compounds **1**–**7** on NO production in LPS-stimulated BV-2 microglial cells (Fig. 6B). Compounds **2** and **3** significantly reduced NO production at 20 µM compared to the LPS-stimulated control (*p* < 0.001). In contrast, compound **1** exhibited moderate inhibitory activity, whereas compounds **4**–**7** showed negligible effects. These findings were further corroborated by IC₅₀ values (Table 2), where compounds **2** and **3** demonstrated potent NO inhibitory activity with IC₅₀ values of 11.1 ± 0.5 µM and 9.9 ± 0.1 µM, respectively, approaching the efficacy of curcumin (IC₅₀ = 2.7 ± 0.3 µM), a well-documented anti-inflammatory agent. Compound **1**, with an IC₅₀ value of 44.8 ± 4.4 µM, exhibited significantly weaker activity, indicating that specific structural features of compounds **2** and **3** contribute to their enhanced bioactivity.

**Table 2:** IC_50_ values of **1**, **2**, and **3** on nitric oxide production inhibitory activities induced by lipopolysaccharide in microglial BV-2 cells

Compound	IC_50_ (µM) ^a^
1	44.8 ± 4.4
2	11.1 ± 0.5
3	9.9 ± 0.1
Curcumin ^*b*^	2.7 ± 0.3

^*a*^ IC_50_ = concentration that reduces NO production by 50%

^*b*^ Positive control used in this study

**Fig. 7:**
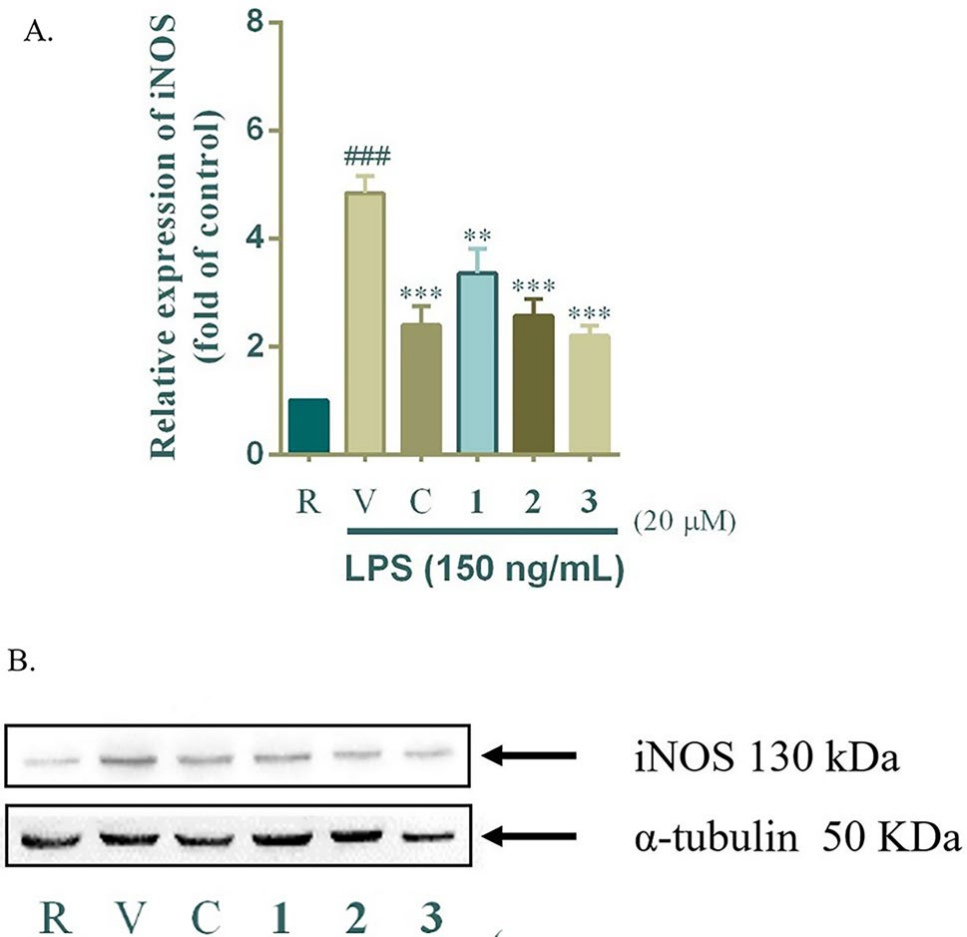
Effects of compounds **1–3** on LPS-induced iNOS expression (**A** and **B**). The concentration of test compounds was 20 µM. Data are expressed as the mean ± SD (*n* = 3). ^#^*p* < 0.05 and ^###^*p* < 0.001, compared with the resting group (R); ^*^p < 0.05, ^**^p < 0.01 and ^***^p < 0.001, compared with the group of stimulation (V)

### Effects of compounds 1–3 on iNOS expression in LPS-stimulated BV-2 cells

Since inducible iNOS is a key enzyme responsible for NO overproduction in inflammatory conditions, the effects of compounds **1**–**3** on iNOS expression were examined using Western blot analysis (Fig. 7). Treatment with compounds **2** and **3** (20 µM) significantly downregulated iNOS expression in LPS-stimulated BV-2 cells (*p* < 0.01 and *p* < 0.001, respectively), while compound **1** exhibited only a mild inhibitory effect. Curcumin, the positive control, showed the strongest inhibition of iNOS expression. These findings indicate that the NO inhibitory activity correlates with iNOS protein suppression and support the role of compounds **2** and **3** as promising iNOS inhibitors, aligning with previous studies that highlight iNOS as a critical target for neuroinflammatory modulation (MacMicking et al. 1997; Mendes et al. 2022).

### Mechanism of compounds 1–3 in interrupting the binding of inducible nitric oxide synthase

**Fig. 8:**
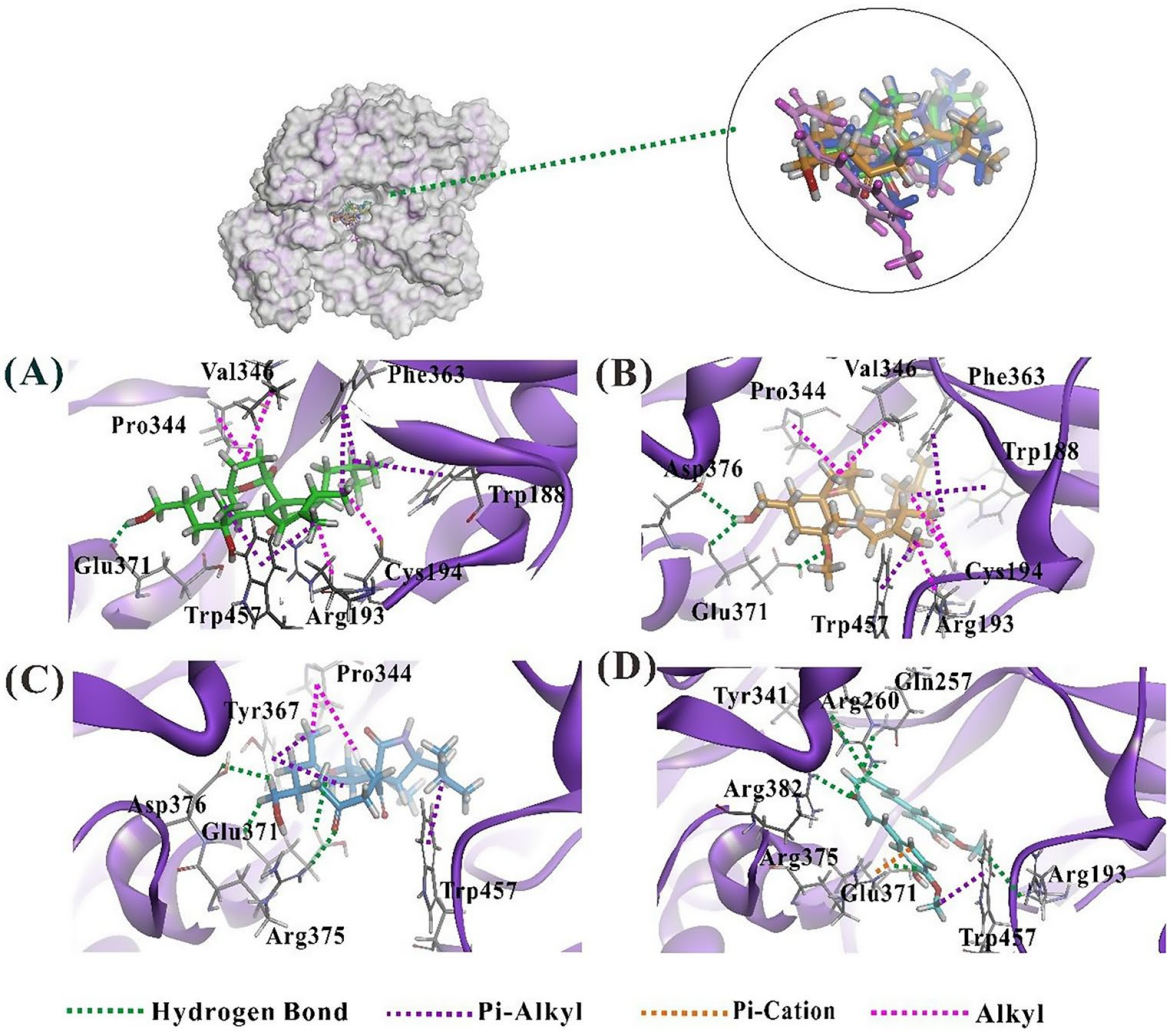
Low-energy binding conformations of compounds **1**(**A**), **2**(**B**), **3**(**C**) and curcumin (**D**) bound to the iNOS (PDB 1QW4) molecular model by computational ligand docking. Key residues are shown as line models background, and hydrogen bonds are labeled as green dashed lines. Pi-alkyl interaction is indicated as purple dashed lines. Pi-cation interaction is indicated as orange dashed lines and Alkyl interaction is indicated as pink dashed lines

Possible molecular interaction mechanism patterns of compounds **1**–**3** with iNOS, particularly focusing on the interaction with the structural amino acid residues in the active cavity important for effective and selective inhibition of NO activity, was elucidated by the molecular docking study. The images of docking conformation of the selected pose for each compound were shown in Fig. 8, and the possible molecular interaction patterns were listed in Table S3.

The results demonstrated that all three compounds interacted with key amino acid residues within the iNOS active site, including Glu371, Pro344, and Trp457. These interactions may block the iNOS active site and consequently inhibit its function. Among them, Compound **3** exhibited the strongest binding affinity (ΔG = -18.8104 kcal/mol) and Cdocker interaction energy at -46.4667 (mol/Kcal), attributed to the formation of a hydrogen bond with catalytic residues Arg375 on the carbonyl groups at C-18 positions of cycloundecene (green dash line, Fig. 8C) and Pi-alkyl interaction with Trp367 (purple dashed lines, Fig. 8C), which significantly enhanced stability in the complex. Curcumin, used as a positive control, showed slightly stronger binding than compound **3**, validating the docking predictions.

In contrast, the binding free energies of compounds **1** and **2** were − 11.3284 kcal/mol and − 15.3685 kcal/mol, respectively (Table S3). Both compounds **1** and **2** displayed alkyl interactions with Phe363 and Trp188, as well as pi-alkyl interactions with Arg193, Cys194, and Val346 (Fig. 8A and B). The critical hydrogen bond formed by compound **3** with Arg375 highlights its higher selectivity and inhibitory potency toward the iNOS active site compared to compounds **1** and **2**.

Moreover, curcumin, as the control compound (ΔG: -19.9625 and CDOCKER interaction energy: -59.3552 kcal/mol), demonstrated significantly higher binding energy and stability relative to the three compounds. Curcumin formed multiple interactions with key residues, including Arg375 and Arg382, via hydrogen bonds, pi-cation interactions, and pi-alkyl interactions thus providing a better reference standard. These extensive interactions contributed to its superior binding performance compared to the tested compounds.

Furthermore, CDOCKER binding energy and △G of compounds **1**, **2** and **3** have the same trend as their inhibited microglial iNOS expression stimulated by LPS method, the later supported the molecular docking predictions were reliable. Previous literature has reported that Arg375 plays a key role in stabilizing iNOS inhibitor binding (Fischmann et al. 1999). Notably, the core macrocyclic structure of the westerchalasins bears notable resemblance to other cytochalasin frameworks recently studied in drug discovery. Valli et al. (2022) reported that natural cytochalasins serve as promising scaffolds for targeting neglected tropical diseases, highlighting their structural adaptability and cross-disease therapeutic potential. This reinforces the relevance of our findings and supports further exploration of westerchalasins in neuroinflammatory contexts.

### Proposed biosynthetic pathway of compounds 1–3

According to the literature, compounds **1**–**3** were likely biosynthesized from the core backbone of cytochalasin through further enzymatic modifications. The core skeletons of cytochalasans were generated via PKS–NRPS hybrid biosynthetic pathways, in which a subsequent Diels–Alder cycloaddition served as a key step in assembling the characteristic tricyclic framework (Boettger et al., 2013; Wang et al. 2014). Based on this, the biosynthetic routes of compounds **1** and **2** were proposed to proceed through an intramolecular carbonyl-ene addition, yielding intermediate *a* with a distinctive 7/6 fused-ring system (Scheme 1). Unlike compound **1**, the isoindolone core backbone underwent epoxidation, elimination, and oxidation to give compound **3** (Scheme 1).

**Fig. 9:**
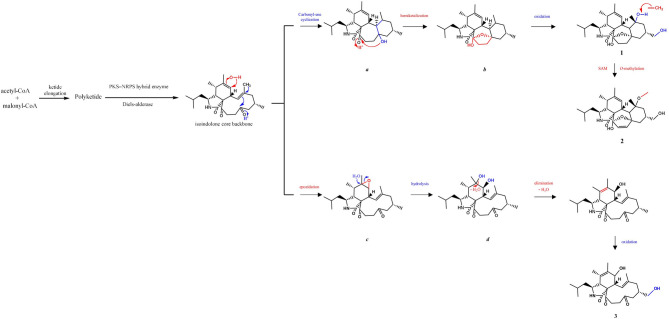
Proposed biosynthetic pathways of compounds **1**–**3**

## Conclusions

The results demonstrated that novel cytochalasins westerchalasin B (**2**) and westerchalasin C (**3**) exhibited significant anti-inflammatory activity by inhibiting NO production and downregulating iNOS expression, suggesting their potential as lead molecules for the development of anti-inflammatory adjunctive therapies. Additionally, molecular docking analysis of the ΔG binding energies indicated that compound **3** exhibited low binding energies, suggesting a strong affinity for binding to the iNOS protein. These results highlighted compound **3** as a promising candidate for neuroprotective drug development. Specifically, compound **3** formed a hydrogen bond with the Arg375 residue, demonstrating higher selectivity and inhibitory potency. Consistent with these findings, compound **3** was considered a promising candidate for the treatment of neurodegenerative diseases, providing a new direction for future drug development.

## Methods

### General experimental procedures

The optical rotations were measured on a JASCO P-2000 Digital Polarimeter (JASCO, Tokyo, Japan). The UV spectra were recorded on a Thermo UV-Visible Heλios α Spectrophotometer (Thermo Scientific, Waltham, MA, USA). The IR spectra were obtained on a JASCO FT/IR 4100 spectrometer (JASCO, Tokyo, Japan). The NMR spectra were recorded at 500 and 125 MHz for ^1^H and ^13^C, respectively, on Bruker AVI 500 MHz FT-NMR spectrometer (Bruker BioSpin GmbH, Ettlingen, Germany). HRESIMS spectra were determined on a Q Exactive ^TM^ Plus Hybrid Quadrupole-Orbitrap Mass Spectrometer (Thermo Fisher Scientific, Bremen, Germany). HPLC separation was performed on a Hitachi HPLC system coupled with a Bischoff RI-8120 RI Detector (Bischoff, Leonberg, Germany) and the Phenomenex Luna column (5 μm PFP column, 100 Å, 250 × 10.0 mm) (Torrance, CA, USA), SunFire column (5 μm C_18_ column, 100 Å, 250 × 10.0 mm) (Milford, Mass, USA) and Phenomenex Gemin column (5 μm C_18_ column, 110 Å, 250 × 4.6 mm) (Torrance, CA, USA). Open column chromatography was performed with Sephadex LH-20 (Amersham Bioscience, Uppsala, Sweden). TLC was carried out with precoated silica gel 60 F254 (Merck, Darmstadt, Germany). Compounds were detected by UV and 10% aqueous H_2_SO_4_ spraying reagent followed by heating at 105 °C for 1 min. The solvents were analytical grade MeOH (Merck, Darmstadt, Germany) for HPLC. ECD data were acquired with a J-815 Circular Dichroism Spectrometer (JASCO, Tokyo, Japan).

### Fungal strain and culture

The fungal strain of *W. dispersa* Ca4-13 was isolated from edible oysters *Crassostrea angulata* collected from Yunlin County, Taiwan, in Nov 2021. The strain was identified based on morphological characteristics and the molecular biology method by amplifying the D1R/D3Ca gene sequence. The sequence data for this strain have been deposited in GenBank with the accession number PQ005631. For cultivation, the purified strain was grown on a CMA medium plate (Becton, Dickinson and Company, Sparks, MD, USA) at 26 °C for 14 days. Agar cultures were cut into small pieces (approximately 0.5 × 0.5 × 0.5 cm^3^) and inoculated into six 2000 mL flasks, each containing 100 mL medium (containing 2 g dextrose, 0.4 g peptone in 50 mL distilled water and 50 mL seawater), along with 200 g brown rice for solid-state fermentation. The fermentation process was carried out at 26 °C for 30 days.

### Extraction and isolation of secondary metabolites

The solid-state fermented product (1800 g, including 1200 g of brown rice and 600 g of PDB medium) was lyophilized, ground into powder, and extracted with 1 L of MeOH three times. The combined methanolic extracts were evaporated into brown residue (16.4 g), which was suspended in 500 mL of H_2_O and partitioned with 500 mL of *n*-hexane three times, then partitioned with 500 mL of ethyl acetate three times. Ethyl acetate layer was concentrated under vacuum to dryness (3.1 g). Subsequently, the ethyl acetate extract was redissolved in 15 mL of methanol and applied onto a Sephadex LH-20 column (2.5 cm i.d. × 56 cm) eluted with MeOH obtain five main fractions I–V based on TLC analyses. Fraction III was further purified by semipreparative HPLC using Phenomenex Luna PFP column with 65% MeOH/H_2_O containing 0.1% formic acid (2.0 mL/min) as eluent to obtain **1** (25.3 mg, *t*_*R*_ = 32.5 min), **2** (6.2 mg, *t*_*R*_ = 34.6 min), **3** (7.0 mg, *t*_*R*_ = 14.5 min) and **4** (56.2 mg, *t*_*R*_ = 22 min). The same fraction was purified using the same column with 70% MeOH/H_2_O containing 0.1% formic acid (2.0 mL/min) as mobile phase to afford **5** (13.6 mg, *t*_*R*_ = 28.5 min), and with 75% MeOH/H_2_O containing 0.1% formic acid (2.0 mL/min) as eluent to give **6** (4.8 mg, *t*_*R*_ = 28.0 min) and **7** (10.2 mg, *t*_*R*_ = 13.6 min).

*Westerchalasin A (****1****)*: colorless crystal; [α]27 D -11.6(*c* 0.1, MeOH); ECD (MeOH): λ_max_ (Δε) 222 (+ 40.6), 205 (–29.8) nm; UV (MeOH) *λ*_max_ (log *ε*) 203 (0.91) nm; HRESIMS *m*/*z* 434.2907 (calcd 434.2901 for C_25_H_40_NO_5_) (Fig. S1); IR (ATR) *ν*_max_ 3347, 1646, 1446, 1288, 1130, 1022, cm^− 1^ (Fig. S2); ^1^H NMR data (methanol-*d*_4_, 500 MHz); ^13^C NMR data (methanol-*d*_4_, 125 MHz) see Table 1.

*Westerchalasin B (****2****)*: whitish powder; [α]27 D -14.7 (*c* 0.1, MeOH); ECD (MeOH): λ_max_ (Δε) 221 (+ 22.7), 205 (–11.1) nm; UV (MeOH) *λ*_max_ (log *ε*) 202 (0.66) nm; HRESIMS *m*/*z* 448.3059 (calcd 438.3057 for C_25_H_40_NO_5_) (Fig. S9); IR (ATR) *ν*_max_ 3679, 3343, 2942, 2345, 1666, 1346, 1037 cm^− 1^ (Fig. S10); ^1^H NMR data (methanol-*d*_4_, 500 MHz); ^13^C NMR data (methanol-*d*_4_, 125 MHz) see Table 1.

*Westerchalasin C (****3****)*: whitish powder; [α]27 D -12.4 (*c* 0.1, MeOH); ECD (MeOH): λ_max_ (Δε) 213 (+ 24.5), 232 (–8.2) nm; UV (MeOH) *λ*_max_ (log *ε*) 204 (1.45) nm; HRESIMS *m*/*z* 432.2746 (calcd 432.2744 for C_25_H_40_NO_5_) (Fig. S17); IR (ATR) *ν*_max_ 3343, 2931, 1689, 1396, 1245, 1103, 1041 cm^− 1^ (Fig. S18); ^1^H NMR data (methanol-*d*_4_, 500 MHz); ^13^C NMR data (methanol-*d*_4_, 125 MHz) see Table 1.

### Single crystal X-ray diffraction analysis

Colorless needle crystals of compound **1** were obtained in methanol/acetone (4:1, v/v). The data collection was carried out using Cu Ka radiation, and the crystal data and experimental details are listed in Tables S1 and S2. Crystallographic data for compound **1** have been deposited in the Cambridge Crystallographic Data Centre (CCDC) with number 2,379,340.

### Cell culture

The mouse microglial BV-2 cell line was cultured as described previously (Hsiao et al. 2020). Before experiments, cells were changed to 0.5% FBS media. Thereafter, cells were treated with vehicle or the indicated concentration of compounds **1**–**7** for 30 min and then stimulated with LPS (L2880, Sigma-Aldrich, St. Louis, US) (150 ng/mL) for 24 h.

### Cell viability assay

The cytotoxicity of compounds addressed in this study against the mouse microglial BV-2 cell line. The cell viability studies were determined by the MTT ([3-(4,5-dimethylthiazol-2-yl)-2,5-diphenyltetrazolium bromide, Sigma-Aldrich, St. Louis, US) method. Cells were seeded in 24-well plates at 5 × 10^5^ cells per well and grown for 24 h before use. The seeded cells were first treated with test compounds at 20 µM for 24 h. The final concentration of DMSO in the culture medium of the treated cells was adjusted to less than 0.5% (v/v) to prevent a solvent effect. Absorbance at 550 nm was obtained by a microplate reader (MRX). All of the experiments were performed in triplicate (Wu et al. 2022).

### Inhibitory activity of nitric oxide (NO) production

Production of NO was evaluated by measuring the levels of nitrite in a conditioned medium as previously described with some modification (Hsieh et al. 2021). The culture supernatants were allowed to react with reconstituted cofactor solution and reconstituted nitrate reductase solution for 1 h at room temperature in the dark according to the instructions of the Nitrate/Nitrite Colorimetric Assay Kit (Cayman). Absorptions were measured at 550 nm using a microplate reader (MRX). Nitrite concentrations were calculated from the standard solutions of sodium nitrite. Curcumin was used as a positive control (Hsiao et al. 2020).

### Western blot analysis

Western blot analysis was performed as previously described (Chou et al. 2010). Briefly, BV-2 cells were cultured as 80% confluence and changed to serum-free medium for 24 h. Thereafter, cells were treated with DMSO or the isolated compound, then stimulated with LPS (150 ng/mL) for 24 h. The quantitative supernatants from cellular lysates were subjected to SDS-PAGE and electrophoretically transferred onto a polyvinylidene fluoride membrane. After soak in the blocking buffer with 5% dry skim milk overnight, membranes were washed three times and sequentially incubated with primary antibodies (anti-iNOS) and HRP-conjugated secondary antibodies, followed by enhanced chemiluminescence detection. Data of specific protein levels are presented as the relative multiples in relation to the control groups.

### Molecular dockings analysis

The CDOCKER protocol in Discovery Studio 2021 (Accelrys Software, Inc., San Diego, CA, USA) was employed to predict the molecular binding interactions between selected compounds and the iNOS enzyme. The crystal structure of inducible nitric oxide synthase (iNOS; PDB ID: 1QW4) was retrieved from the Protein Data Bank (http://www.rcsb.org/pdb/). The structures of compounds **1**–**3**, along with curcumin as a positive control, were drawn using ChemBioDraw Ultra 13.3, converted into 3D conformations, and energy-minimized using the CHARMm force field with the conjugate gradient method in Discovery Studio (convergence criterion of 0.001 kcal/mol) (Brooks et al. 1983). The protein structure was prepared by removing water molecules, adding hydrogen atoms, and reconstructing missing loop regions using the built-in loop modeling module. Protonation and ionization states were adjusted accordingly, followed by a final energy minimization to optimize the structure for docking. The binding site was identified based on PDB annotations and refined using the Binding Site module. A binding sphere was defined (coordinates: x = 25.5149, y = 70.3256, z = 21.8314; radius = 21.925 Å) to guide ligand placement. Energy-minimized ligand structures were docked into the active site using the CDOCKER algorithm. For each compound, the docking pose with the highest CDOCKER energy (-CDOCKER_INTERACTION_ENERGY) was selected as the optimal conformation. Furthermore, ligand-binding free energies were estimated using the Generalized Born with Molecular Volume (GBMV) method to provide insights into complex stability (Wu et al. 2003; Kelley et al. 2015; Eladl et al. 2024).

### Statistical analysis

All experiments were performed in triplicate (*n* = 3) and the data are presented as means ± standard deviations (SD). Statistical analysis was conducted using the GraphPad Prism version. 9.5.1 (GraphPad Software, San Diego, CA, USA). One-way analysis of variance (ANOVA) followed by Tukey’s post hoc test was used for group comparisons (Yadid et al. 2020). Statistical significance was determined based on the difference between groups, with *p* ≤ 0.05 considered statistically significant.

## Supplementary Information

Below is the link to the electronic supplementary material.

Supplementary Material 1

## Data Availability

Data of this study is available with the first author Ms. Huang.

## Abbreviations

CCDC: Cambridge Crystallographic Data Centre
ECD: Electronic circular dichroism
HMBC: Heteronuclear multiple bond coherence
HPLC: High performance liquid chromatography
HRESIMS: High resolution electrospray ionization mass spectroscopy
HSQC: Heteronuclear single quantum correlation
iNOS: Inducible nitric oxide synthase
IR: Infrared
LPS: Lipopolysaccharides
MeOH: Methanol
MTT: 3-(4,5-Dimethyl-2-thiazolyl)-2,5-diphenyl-2H-tetrazolium Bromide
NMR: Nuclear magnetic resonance
NO: Nitric oxide
NOESY: Nuclear Overhauser effect spectroscopy
PDB: Potato dextrose broth
RI: Refractive index
ROS: Reactive oxygen species
TLC: Thin layer chromatography
