# Supplementary material for: Novel inducible nitric oxide synthase-inhibiting cytochalasins from an oyster-derived fungus Westerdykella dispersa Ca4-13: structural insights and molecular docking analysis
Source: Bot Stud. 2025 Oct 7;66:33. doi: 10.1186/s40529-025-00481-z (PMC12504155; doi:10.1186/s40529-025-00481-z)
Supplement: Supplementary file 1 — Supplementary Material 1 [file 40529_2025_481_MOESM1_ESM.docx]

**Supporting Information**

Novel iNOS-Inhibiting Cytochalasins from an Oyster-Derived Fungus Westerdykella dispersa Ca4-13: Structural Insights and Molecular Docking Analysis

Shu-Jung Huang^1^, Su-Jung Hsu^1^, Shih-Wei Wang^2,3,4^, Yi-Chien Liu^5,6^, Cheng-Yan Jiang^6,7^, George Hsiao^6,7*^, Tzong-Huei Lee^1,8*^

^1^Institute of Fisheries Science, National Taiwan University, 106319, Taipei, Taiwan

^2^Department of Medicine, MacKay Medical College, New Taipei City 25245, Taiwan.

^3^Institute of Biomedical Sciences, Mackay Medical College, New Taipei City 25245, Taiwan.

^4^School of Pharmacy, College of Pharmacy, Kaohsiung 807378, Taiwan

^5^Ph.D. Program in Drug Discovery and Development Industry, College of Pharmacy, Taipei Medical University, Taipei 11031, Taiwan

^6^Department of Pharmacology, School of Medicine, College of Medicine, Taipei Medical University, Taipei 11031, Taiwan.

^7^Graduate Institute of Medical Sciences, College of Medicine, Taipei Medical University, Taipei 11031, Taiwan.

^8^Department of Life Science, College of Life Science, National Taiwan University, 106319, Taipei, Taiwan

*Correspondence: [geohsiao@tmu.edu.tw](https://hub.tmu.edu.tw/zh/persons/george-hsiao) (G.H.); [thlee1@ntu.edu.tw](mailto:thlee1@ntu.edu.tw) (T.-H.L.)

Table of Contents

[Fig. S1. HRESIMS spectrum of compound 1. 3](#_Toc189676460)

[Fig. S2. IR (ZnSe) spectrum of compound 1. 4](#_Toc189676461)

[Fig. S3. ^1^H NMR (500 MHz, methanol*-d*_4_) spectrum of compound 1. 5](#_Toc189676462)

[Fig. S4. ^13^C NMR (125 MHz, methanol*-d*_4_) spectrum of compound 1. 6](#_Toc189676463)

[Fig. S5. HSQC spectrum of compound 1. 7](#_Toc189676464)

[Fig. S6. COSY spectrum of compound 1. 8](#_Toc189676465)

[Fig. S7. HMBC spectrum of compound 1. 9](#_Toc189676465)

[Fig. S8. NOESY spectrum of compound 1. 10](#_Toc189676466)

[Fig. S9. HRESIMS spectrum of compound 2. 11](#_Toc189676467)

[Fig. S10. IR (ZnSe) spectrum of compound 2. 11](#_Toc189676468)

[Fig. S11. ^1^H NMR (500 MHz, methanol*-d*_4_) spectrum of compound 2. 12](#_Toc189676469)

[Fig. S12. ^13^C NMR (125 MHz, methanol*-d*_4_) spectrum of compound 2. 13](#_Toc189676470)

[Fig. S13. HSQC spectrum of compound 2. 14](#_Toc189676471)

[Fig. S14. COSY spectrum of compound 2. 15](#_Toc189676472)

[Fig. S15. HMBC spectrum of compound 2. 16](#_Toc189676473)

[Fig. S16. NOESY spectrum of compound 2. 17](#_Toc189676474)

[Fig. S17. HRESIMS spectrum of compound 3. 18](#_Toc189676475)

[Fig. S18. IR (ZnSe) spectrum of compound 3. 18](#_Toc189676476)

[Fig. S19. ^1^H NMR (500 MHz, methanol*-d*_4_) spectrum of compound 3. 19](#_Toc189676477)

[Fig. S20. ^13^C NMR (125 MHz, methanol*-d*_4_) spectrum of compound 3. 20](#_Toc189676478)

[Fig. S21. HSQC spectrum of compound 3. 21](#_Toc189676479)

[Fig. S22. COSY spectrum of compound 3. 22](#_Toc189676480)

[Fig. S23. HMBC spectrum of compound 3. 23](#_Toc189676481)

[Fig. S24. NOESY spectrum of compound 3. 24](#_Toc189676482)

[Table S1. Crystal data and experimental details for compound 1. 25](#_Toc189676483)

[Table S2. Bond lengths [Å] and angles [°] for compound 1. 2](#_Toc189676484)6

[Table S3. CDOCKER energy and interaction pattern of potential inducible nitric oxide synthase (iNOS) inhibitors predicted by molecular docking analysis. 30](#_Toc189676485)


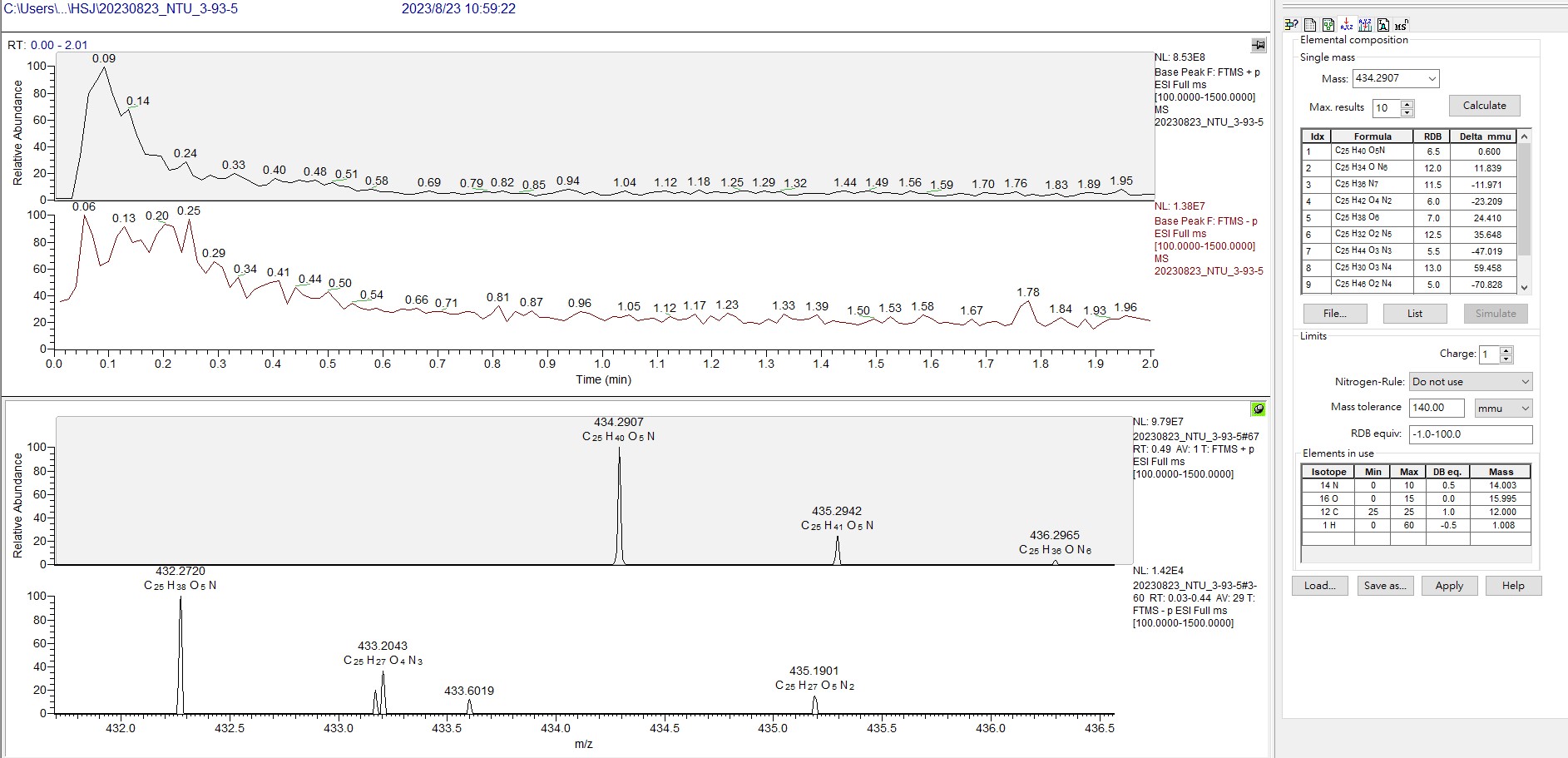


# Fig. S1. HRESIMS spectrum of compound 1.


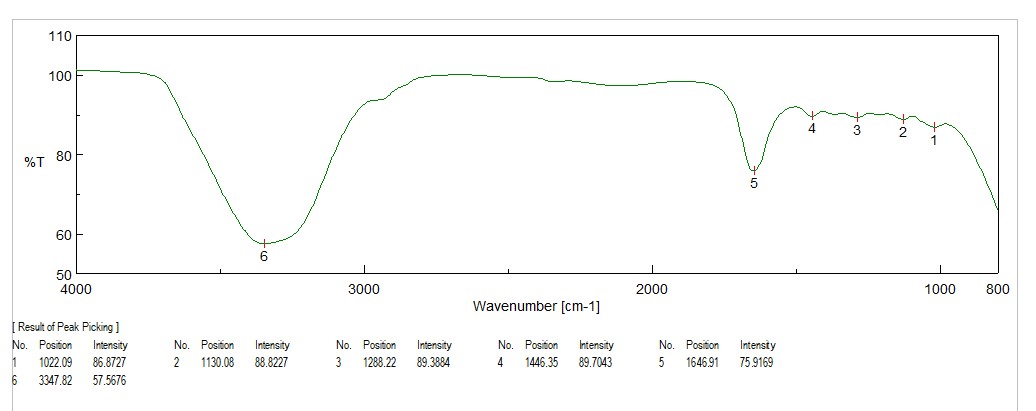


#

# Fig. S2. IR (ZnSe) spectrum of compound 1.


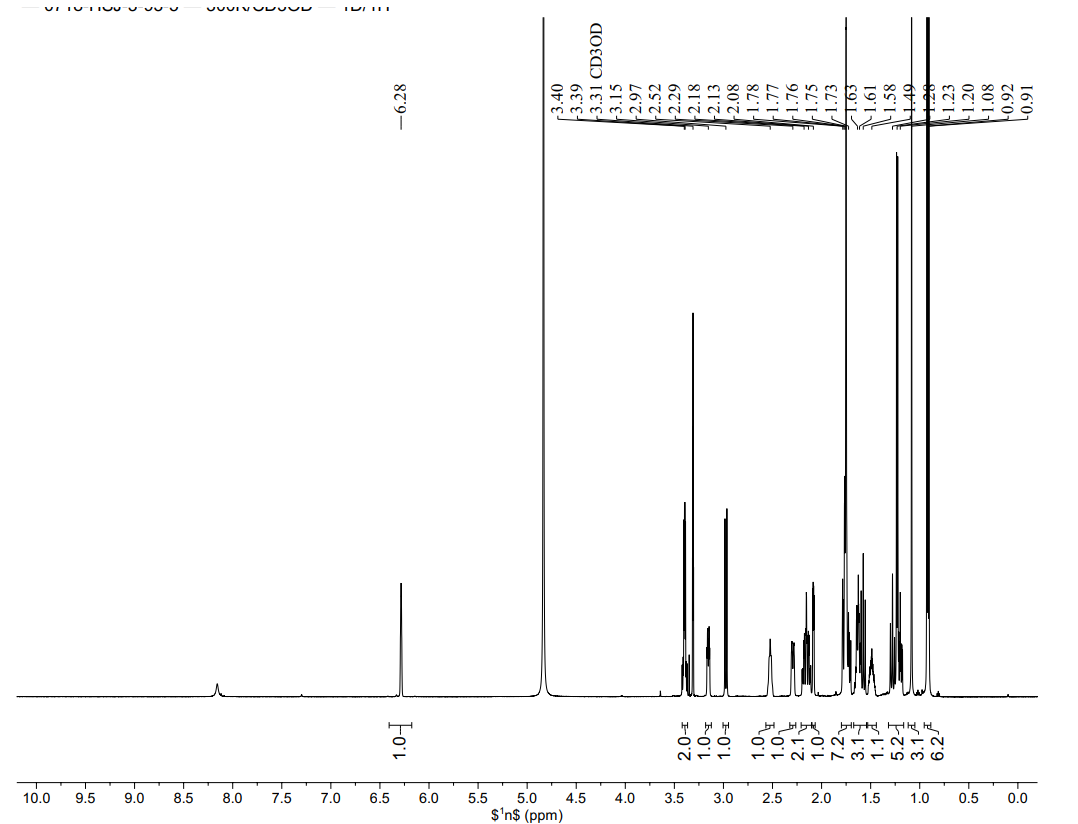

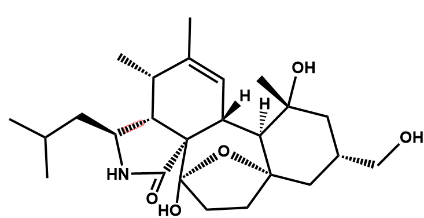


# Fig. S3. ^1^H NMR (500 MHz, methanol*-d*_4_) spectrum of compound 1.


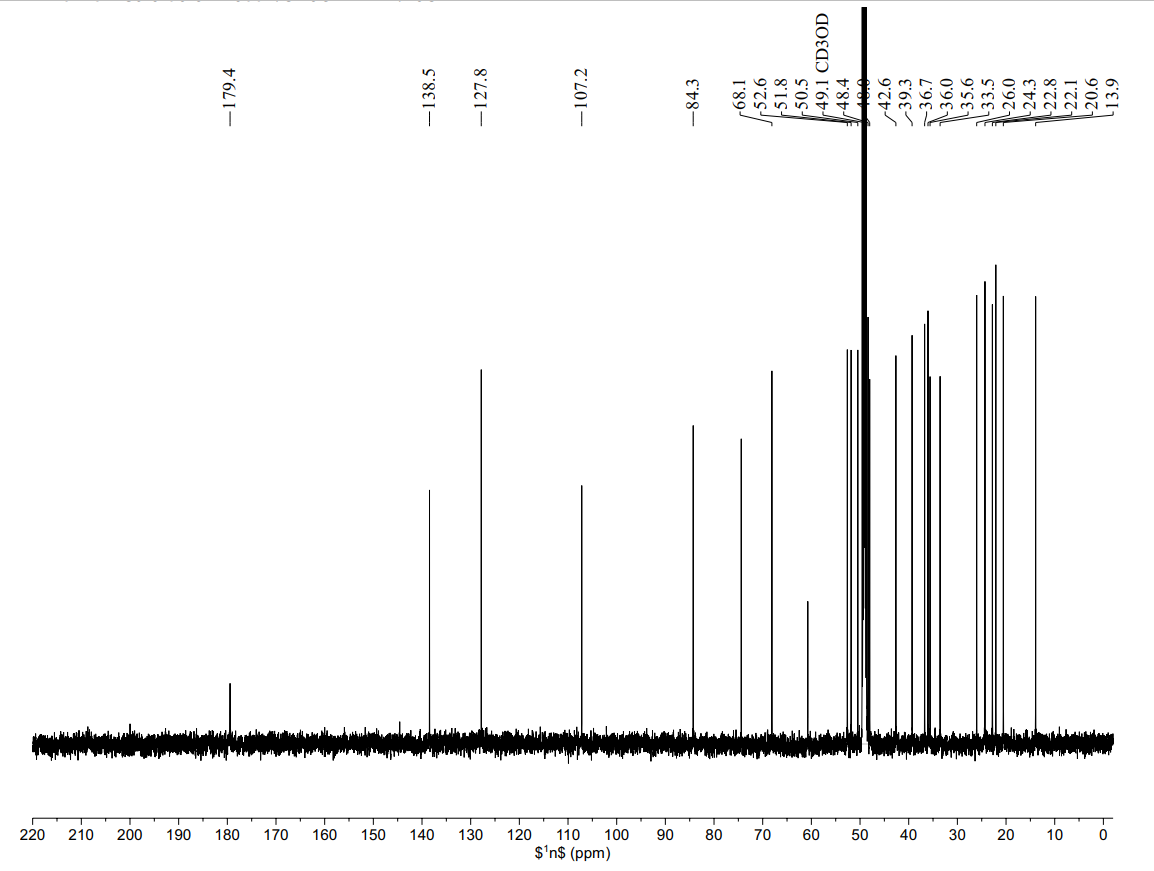

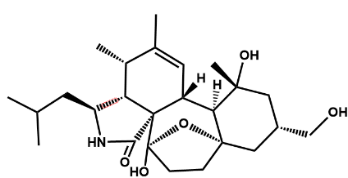


# Fig. S4. ^13^C NMR (125 MHz, methanol*-d*_4_) spectrum of compound 1.


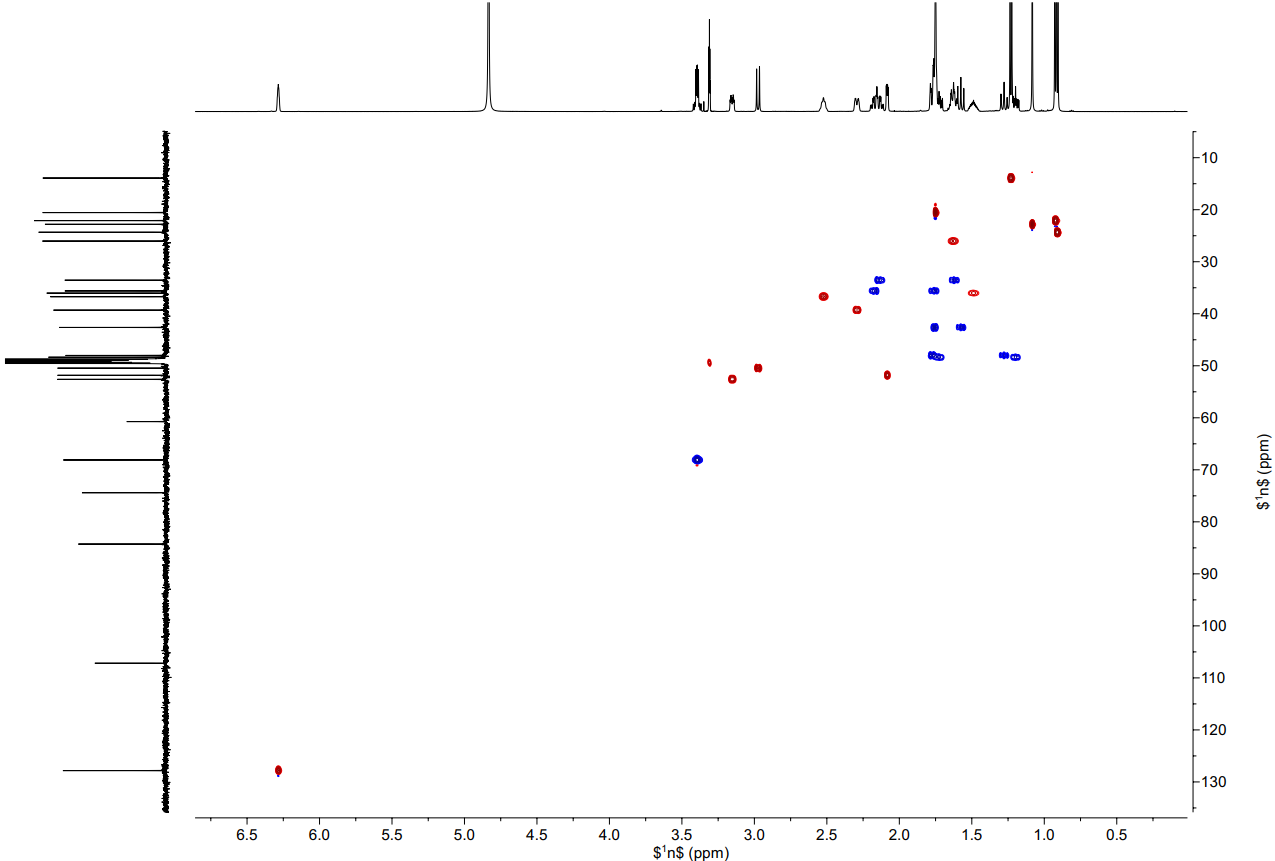

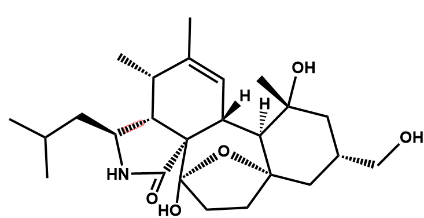


# Fig. S5. HSQC spectrum of compound 1.


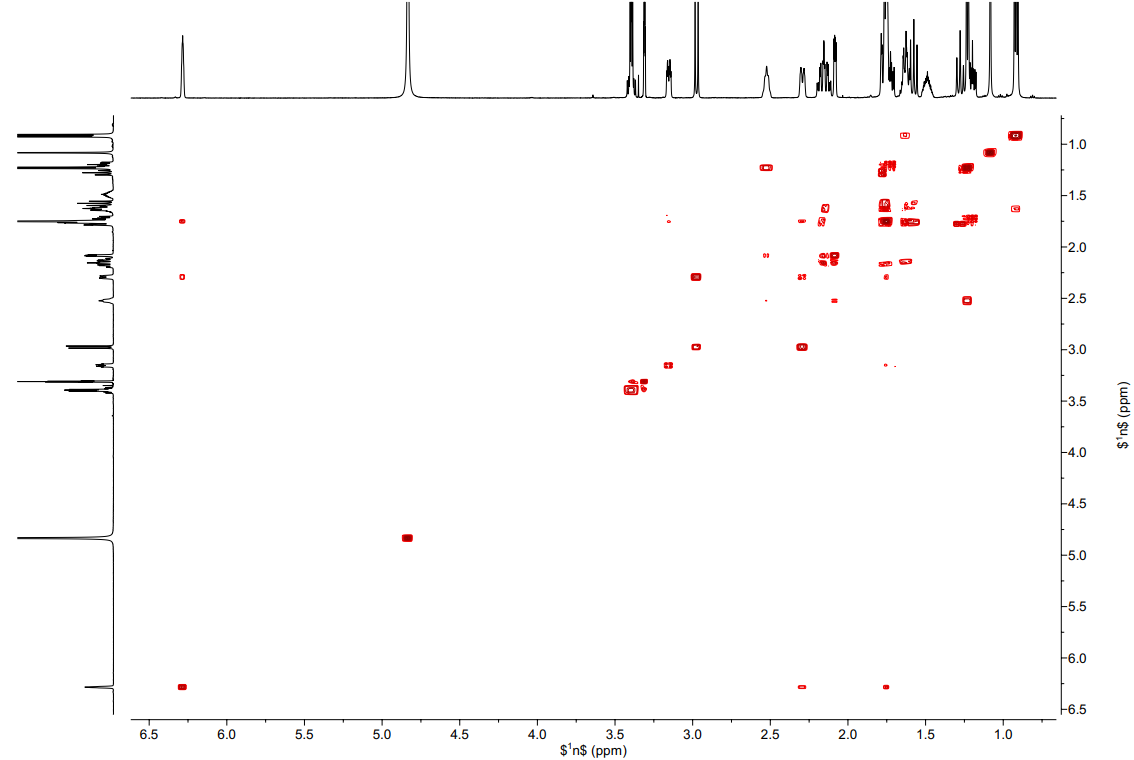

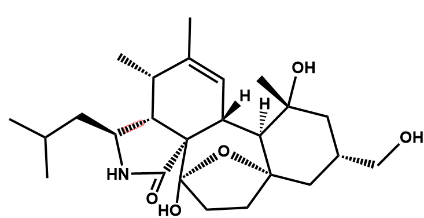


# Fig. S6. COSY spectrum of compound 1.


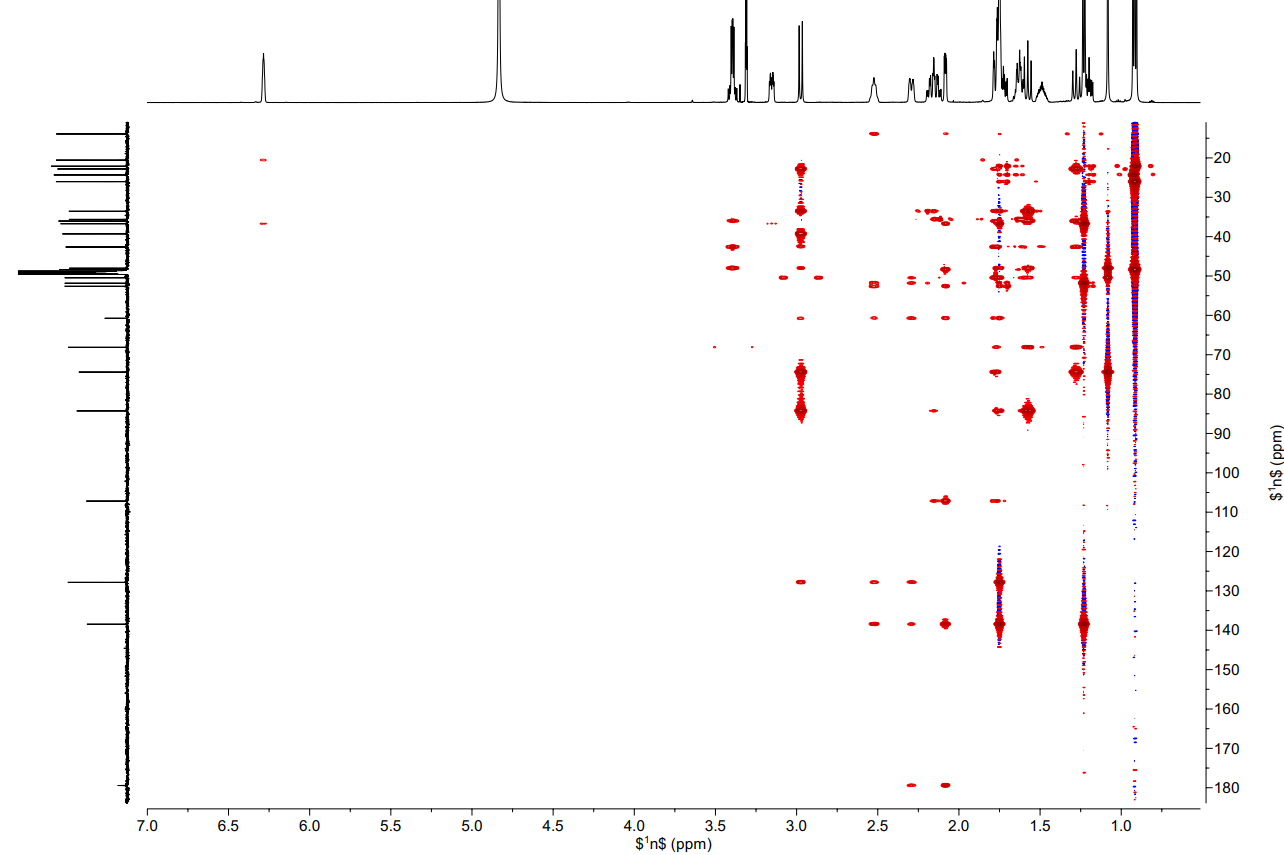

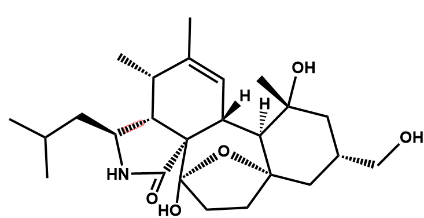


**Fig. S7.** HMBC spectrum of compound **1**.


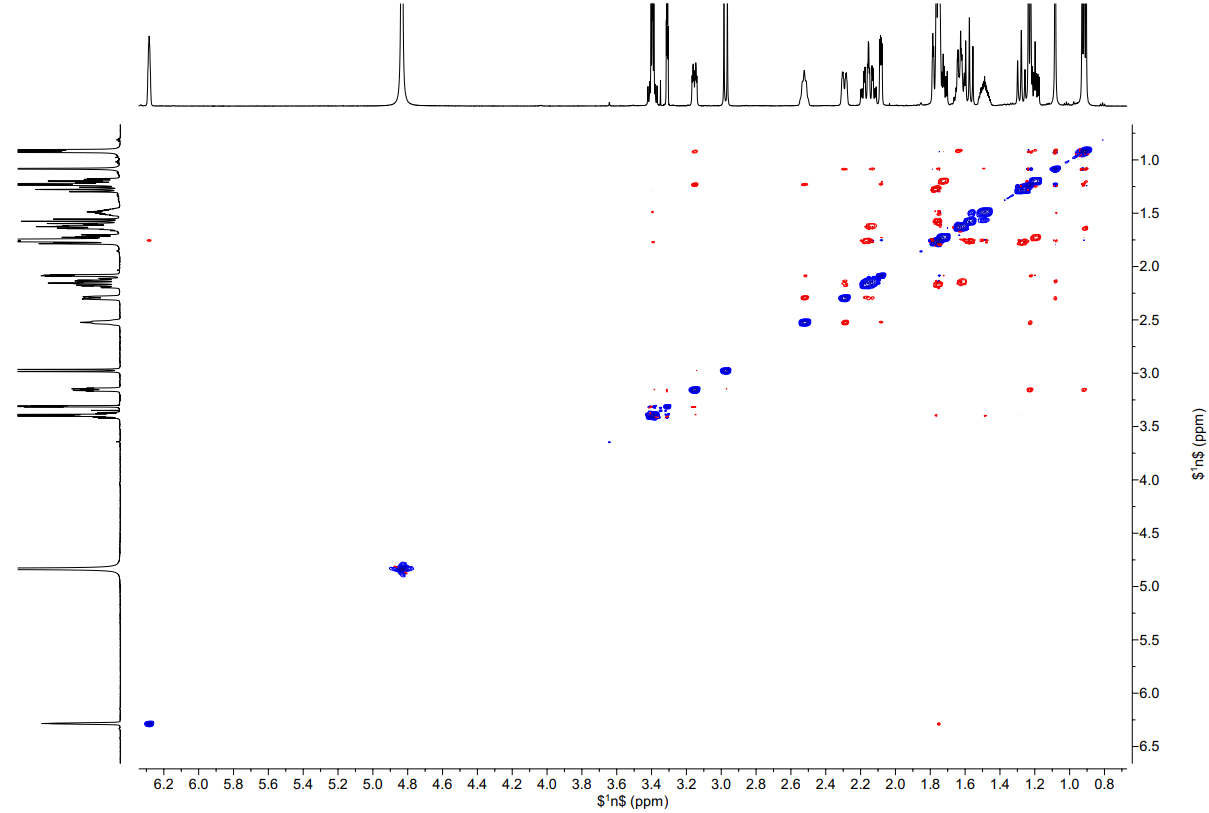

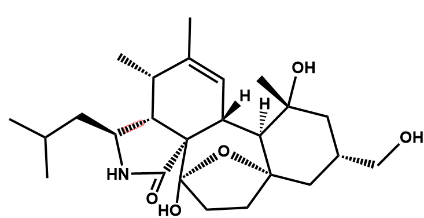


# Fig. S8. NOESY spectrum of compound 1.


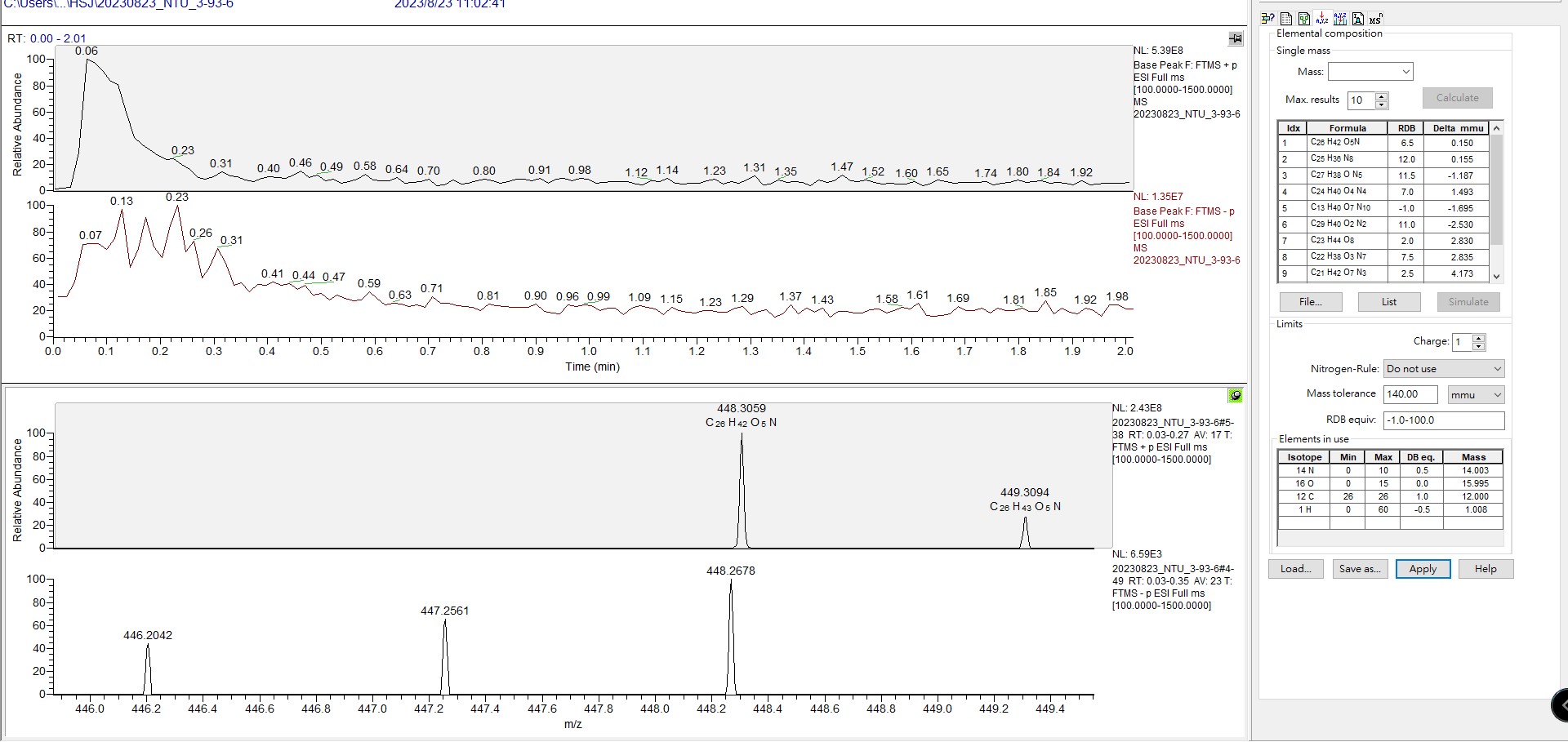


# Fig. S9. HRESIMS spectrum of compound 2.


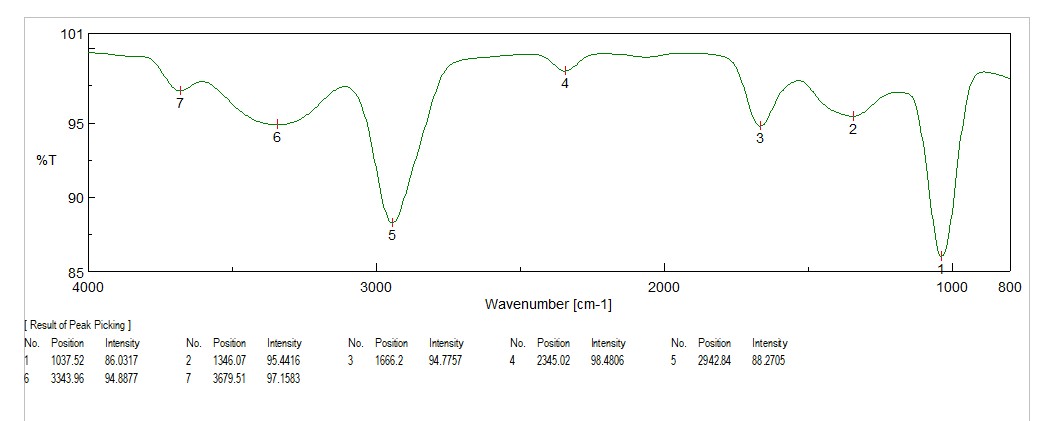


# Fig. S10. IR (ZnSe) spectrum of compound 2.


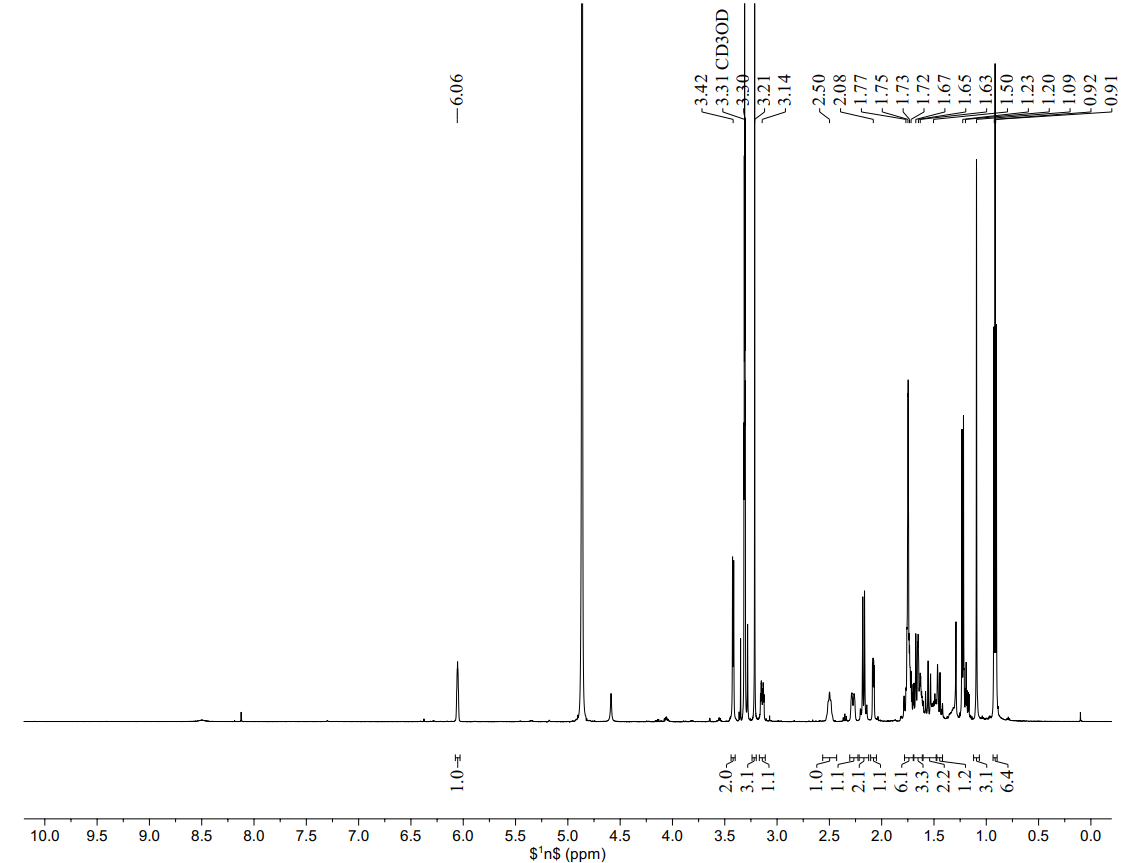

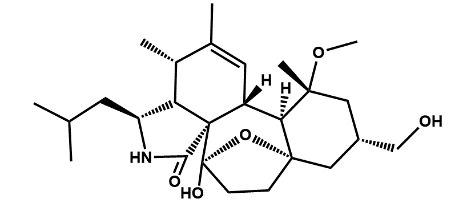


# Fig. S11. ^1^H NMR (500 MHz, methanol*-d*_4_) spectrum of compound 2.


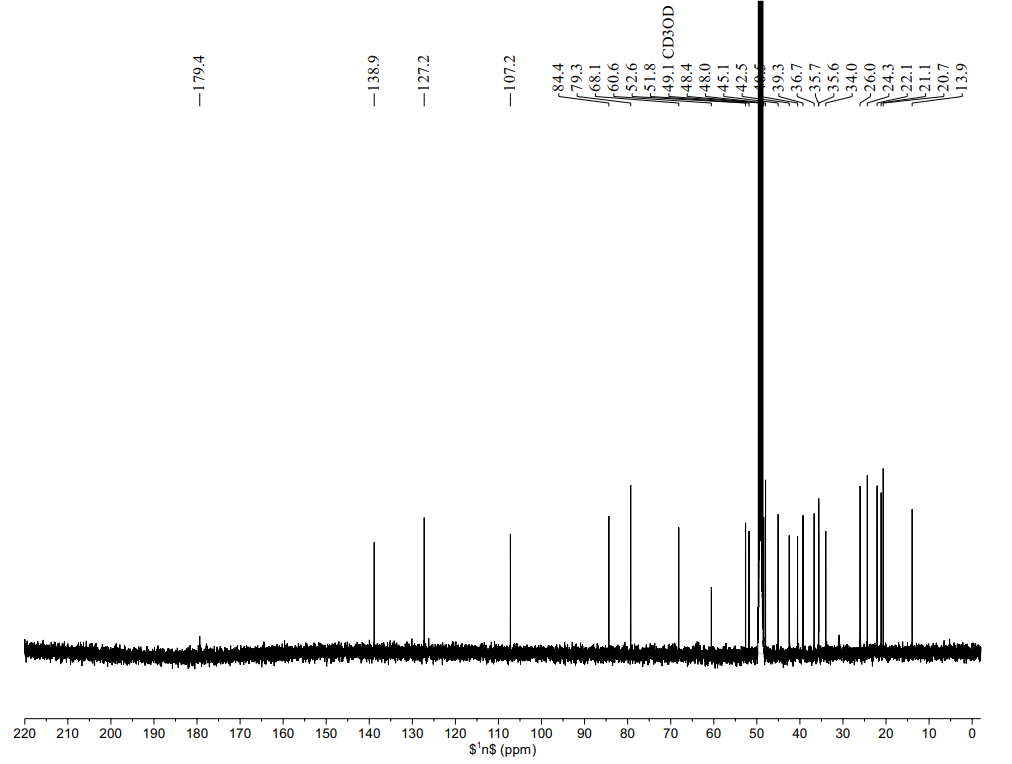

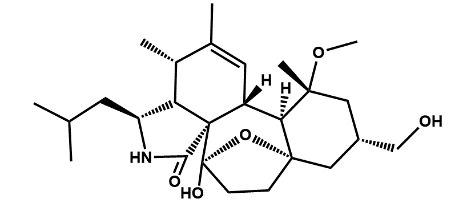


# Fig. S12. ^13^C NMR (125 MHz, methanol*-d*_4_) spectrum of compound 2.


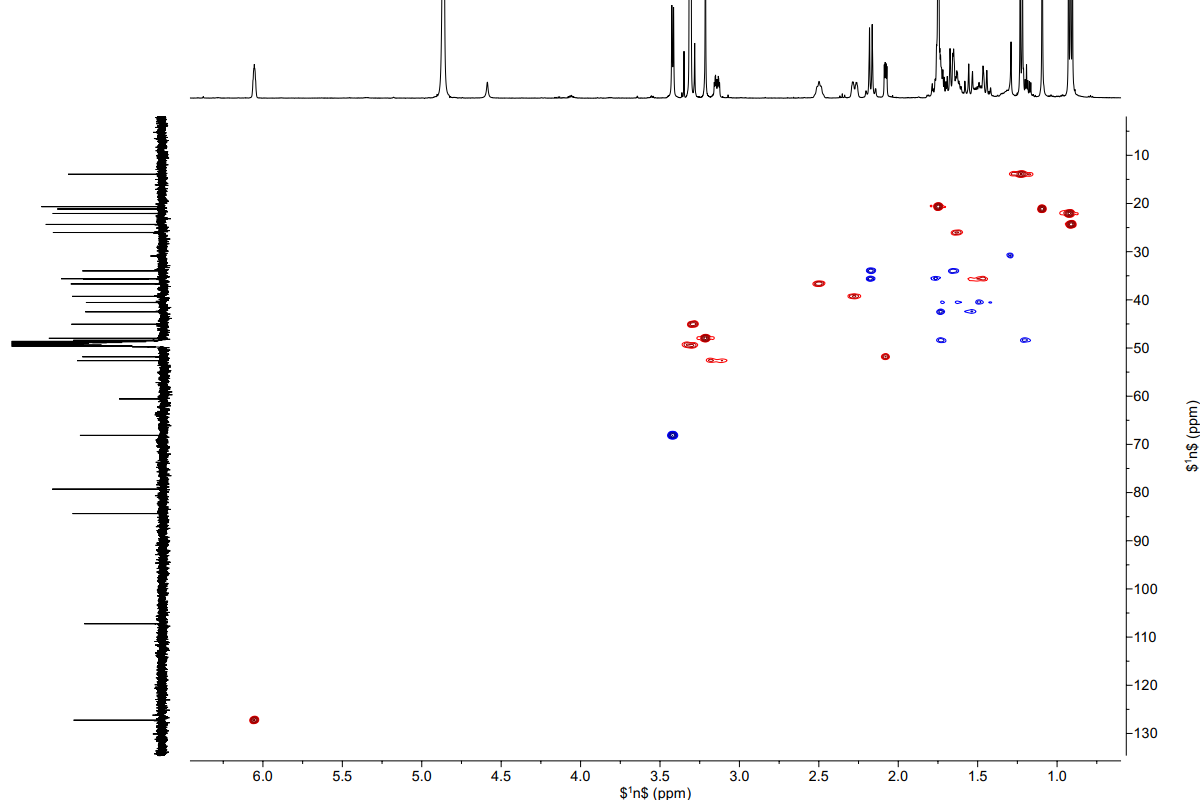

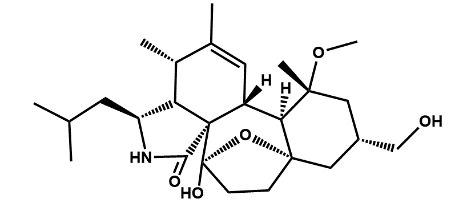


# Fig. S13. HSQC spectrum of compound 2.


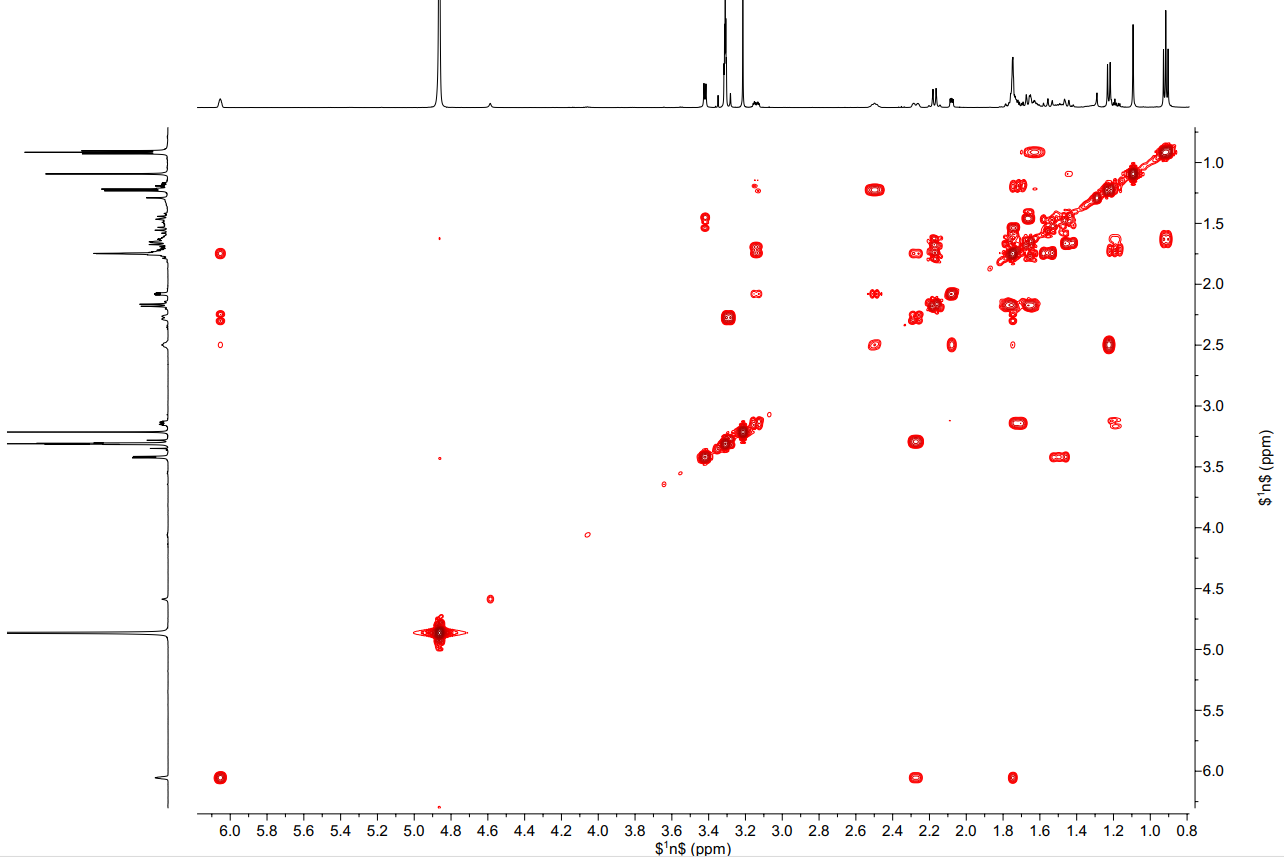

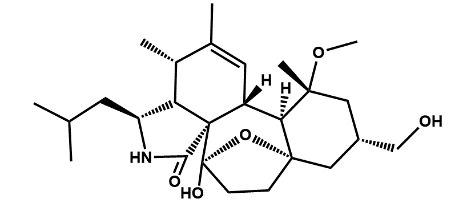


# Fig. S14. COSY spectrum of compound 2.


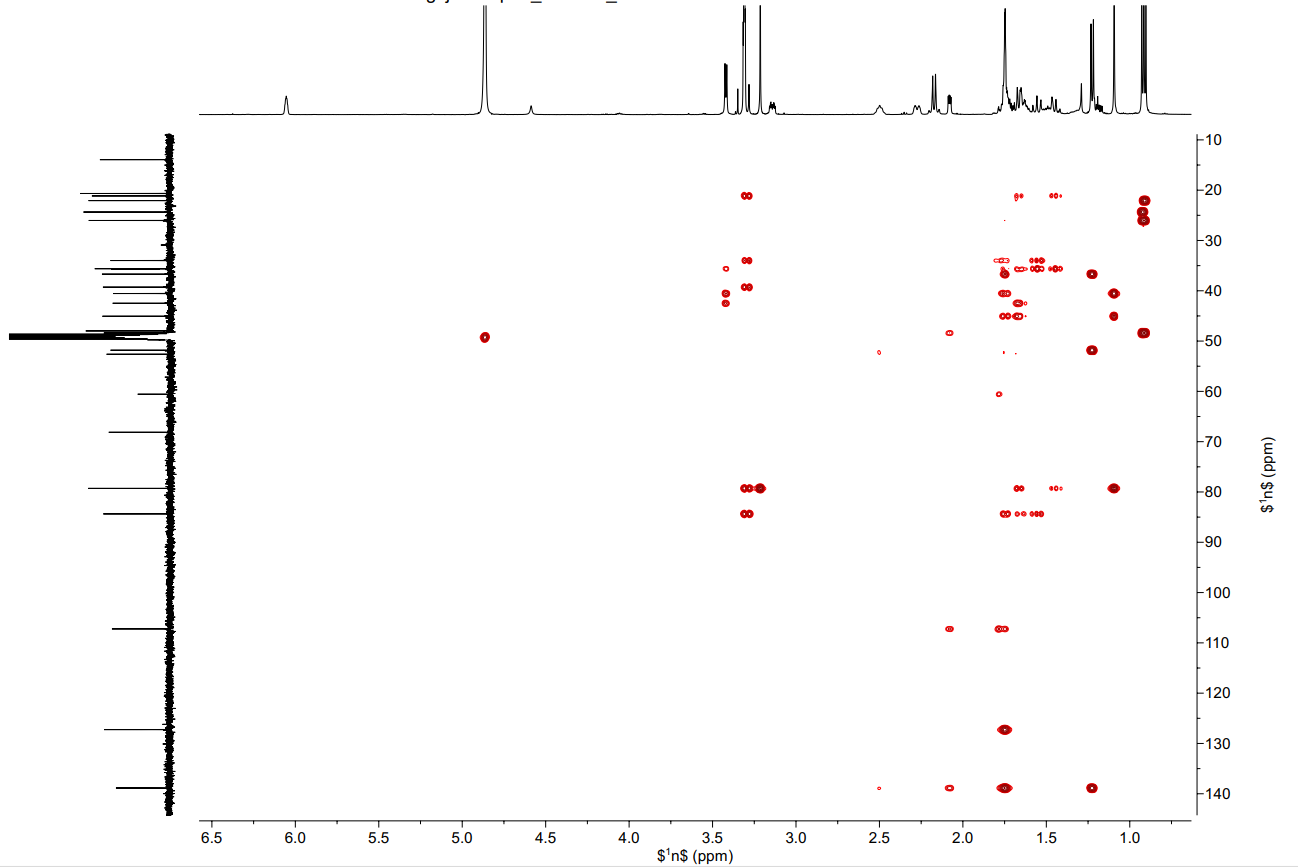

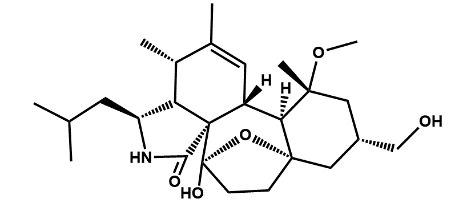


# Fig. S15. HMBC spectrum of compound 2.


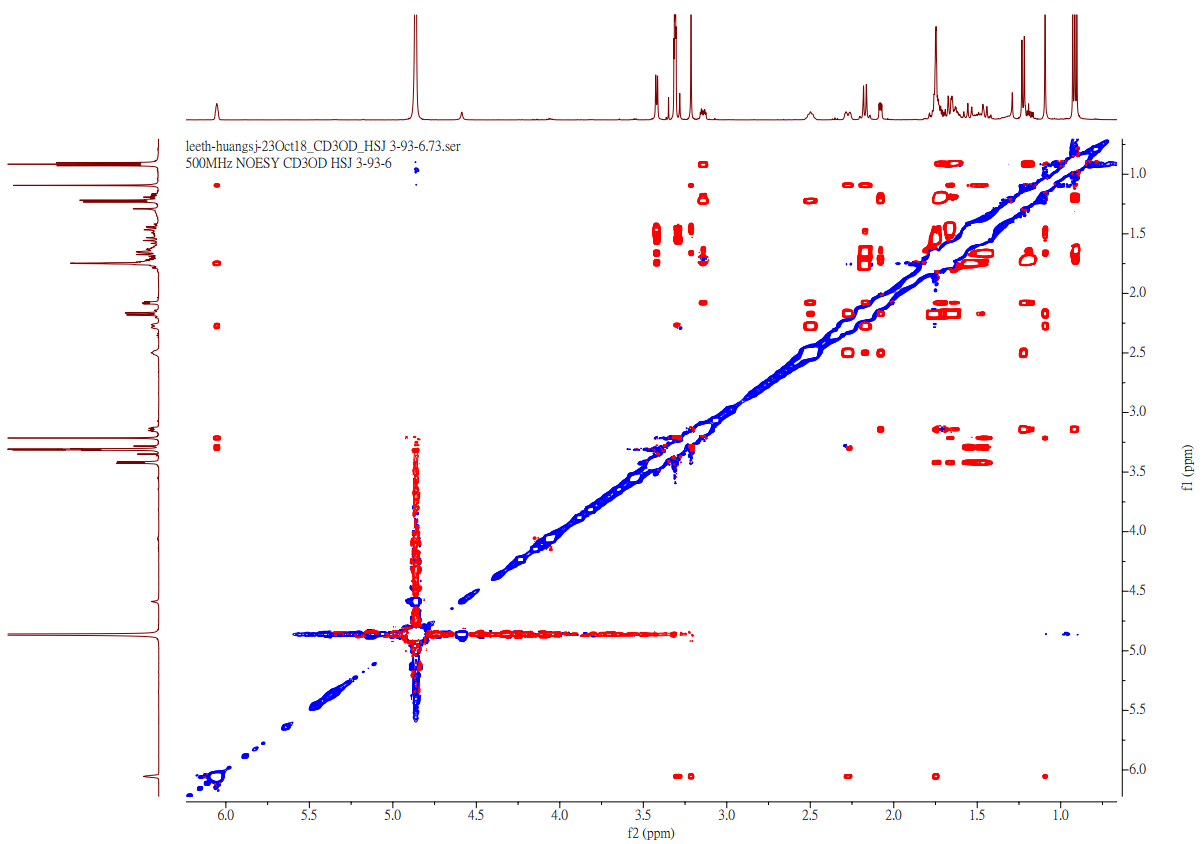

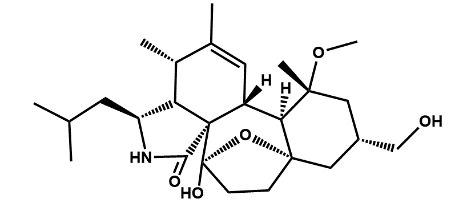


# Fig. S16. NOESY spectrum of compound 2.


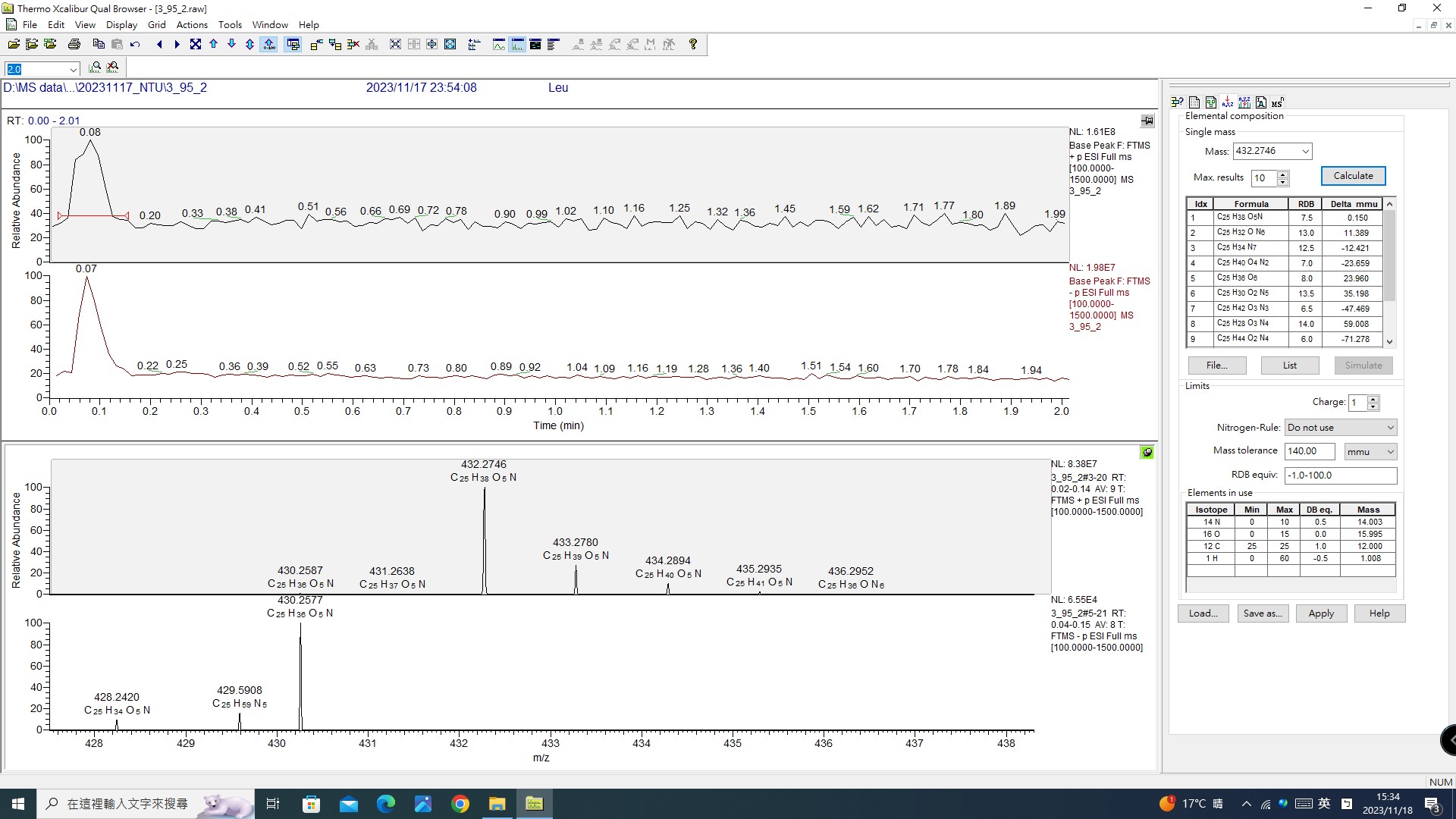


# Fig. S17. HRESIMS spectrum of compound 3.


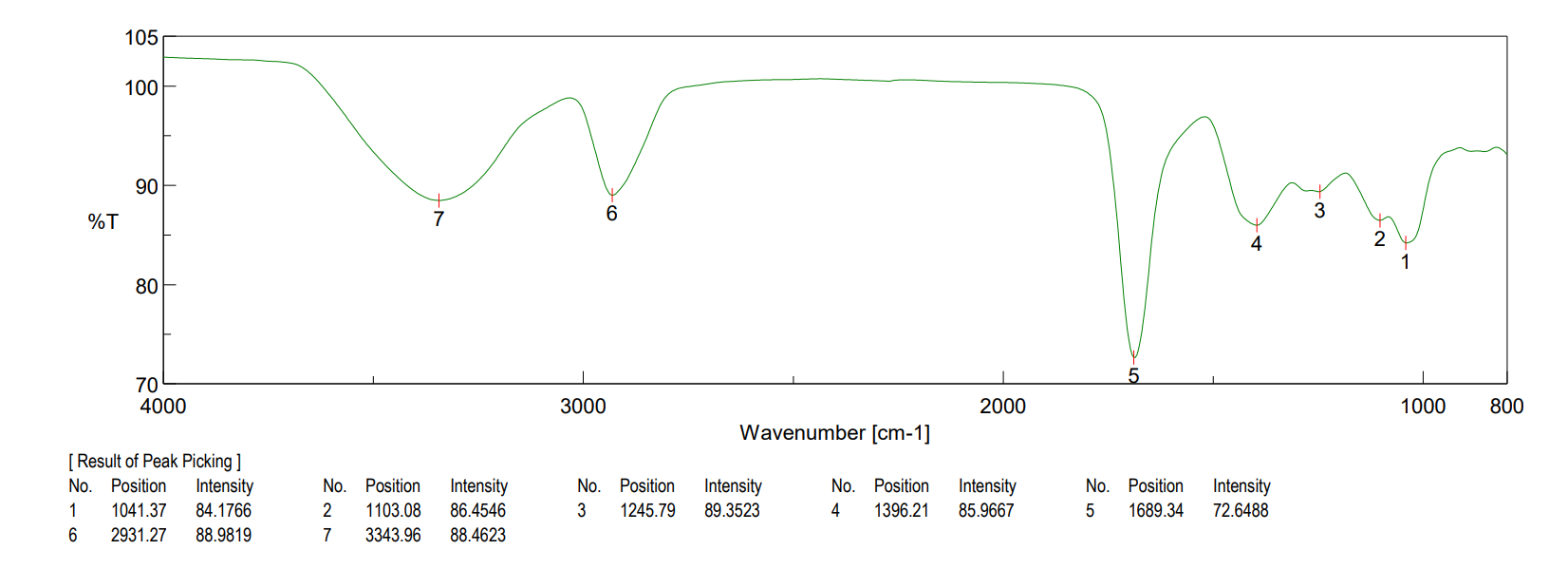


# Fig. S18. IR (ZnSe) spectrum of compound 3.


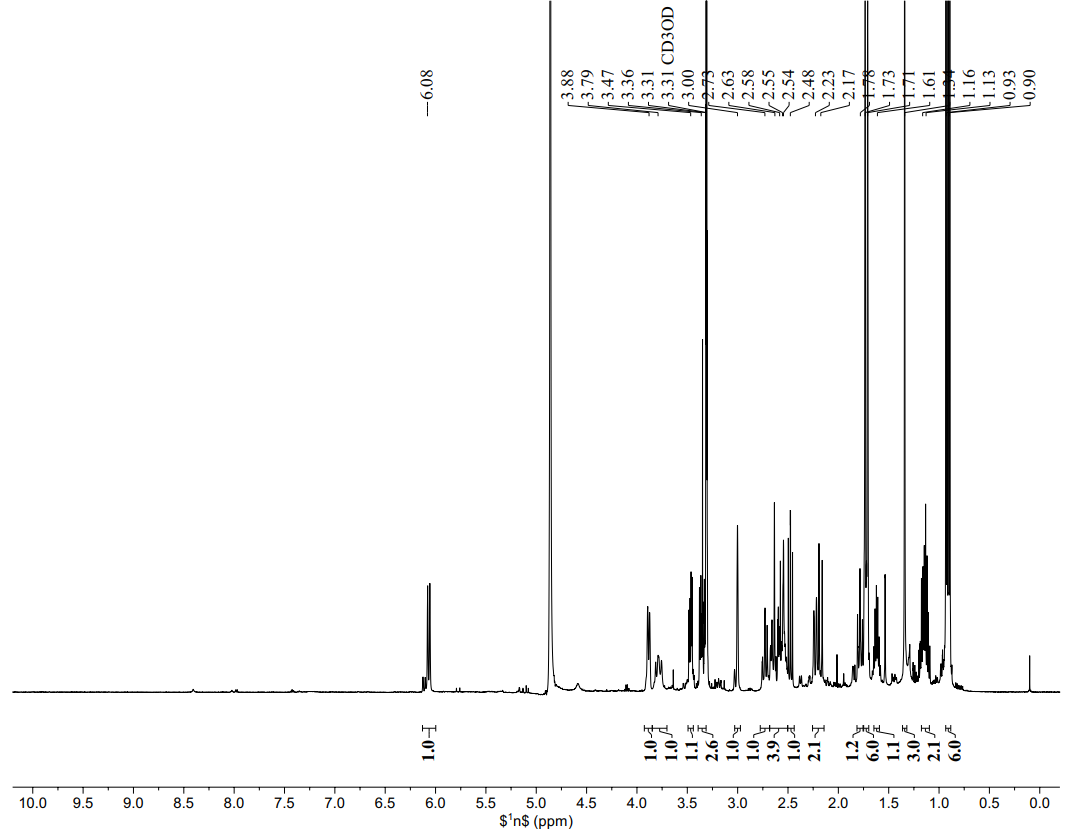

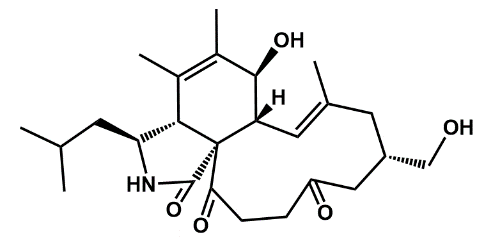


# Fig. S19. ^1^H NMR (500 MHz, methanol*-d*_4_) spectrum of compound 3.


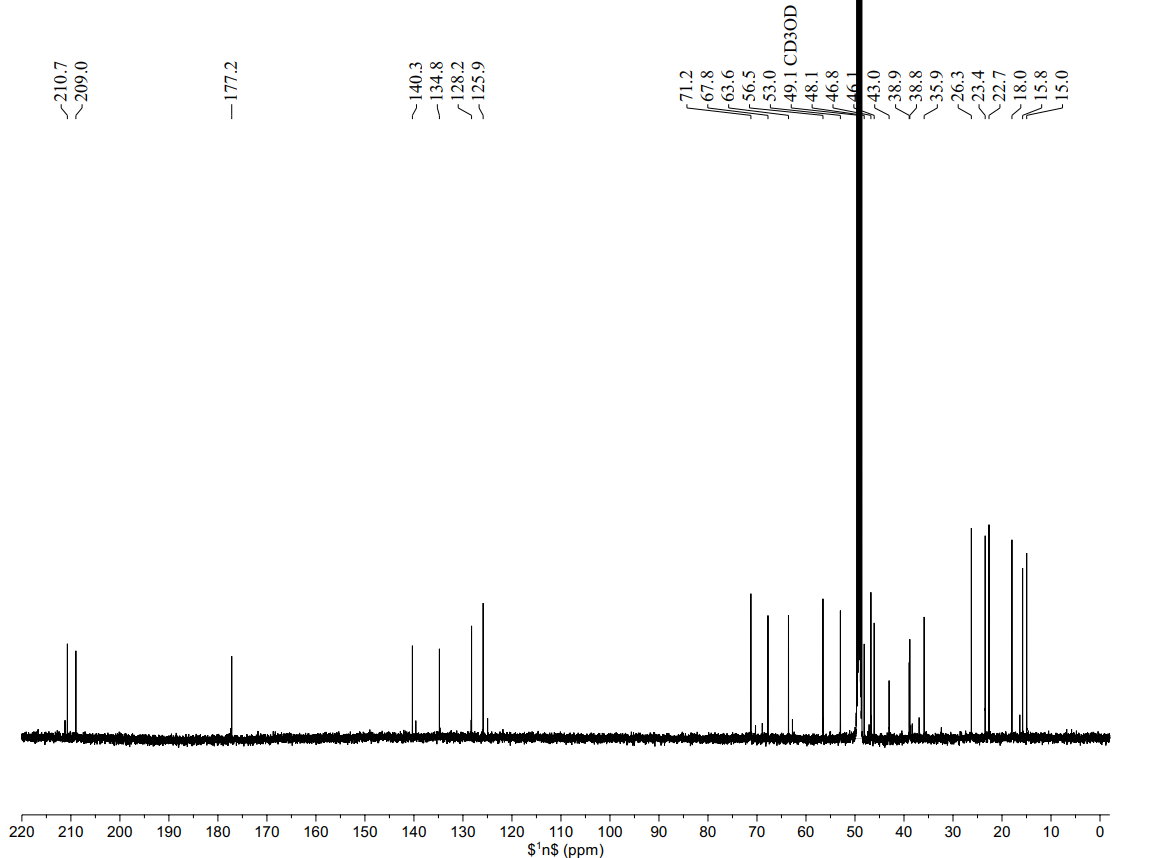

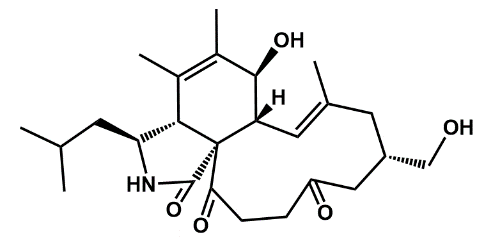


# Fig. S20. ^13^C NMR (125 MHz, methanol*-d*_4_) spectrum of compound 3.


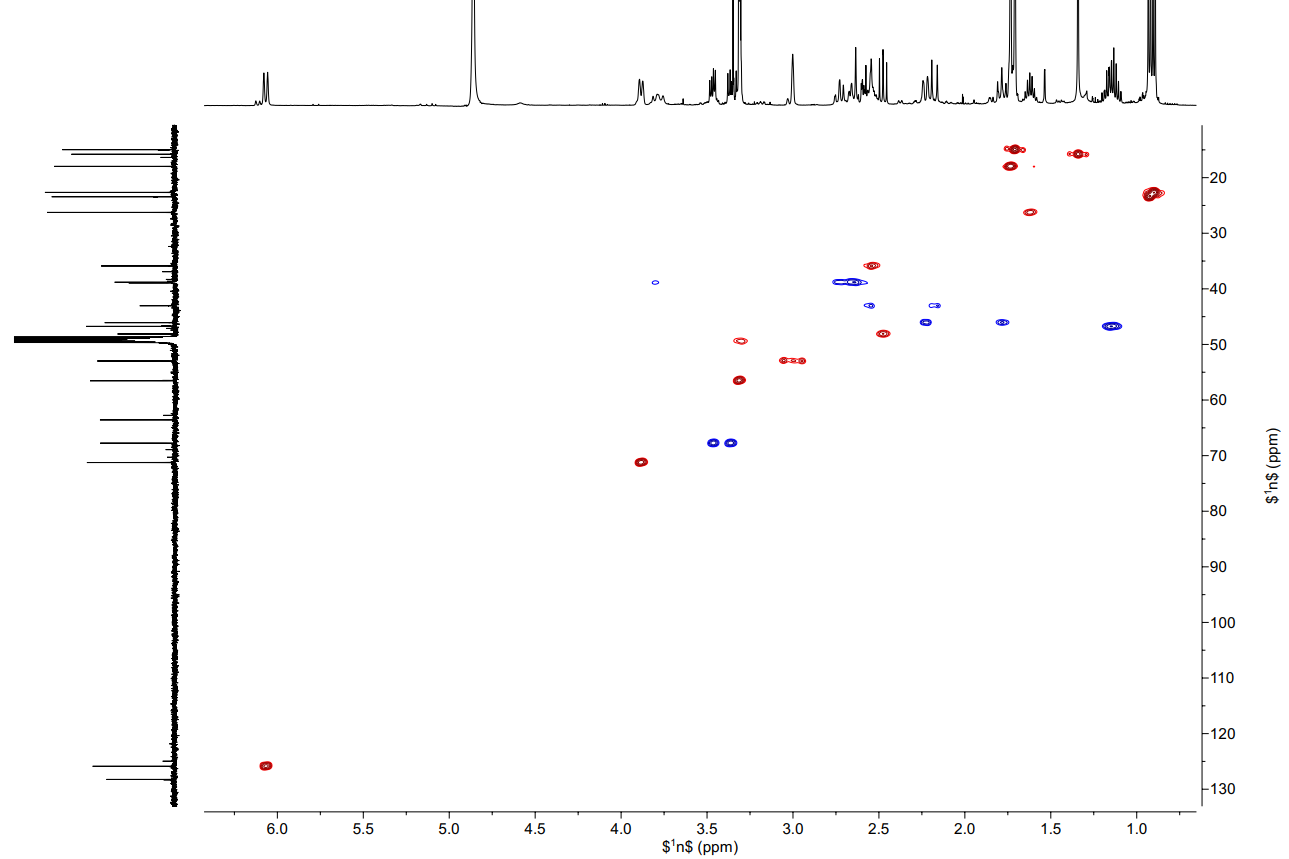

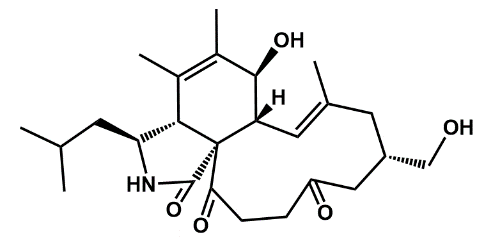


# Fig. S21. HSQC spectrum of compound 3.


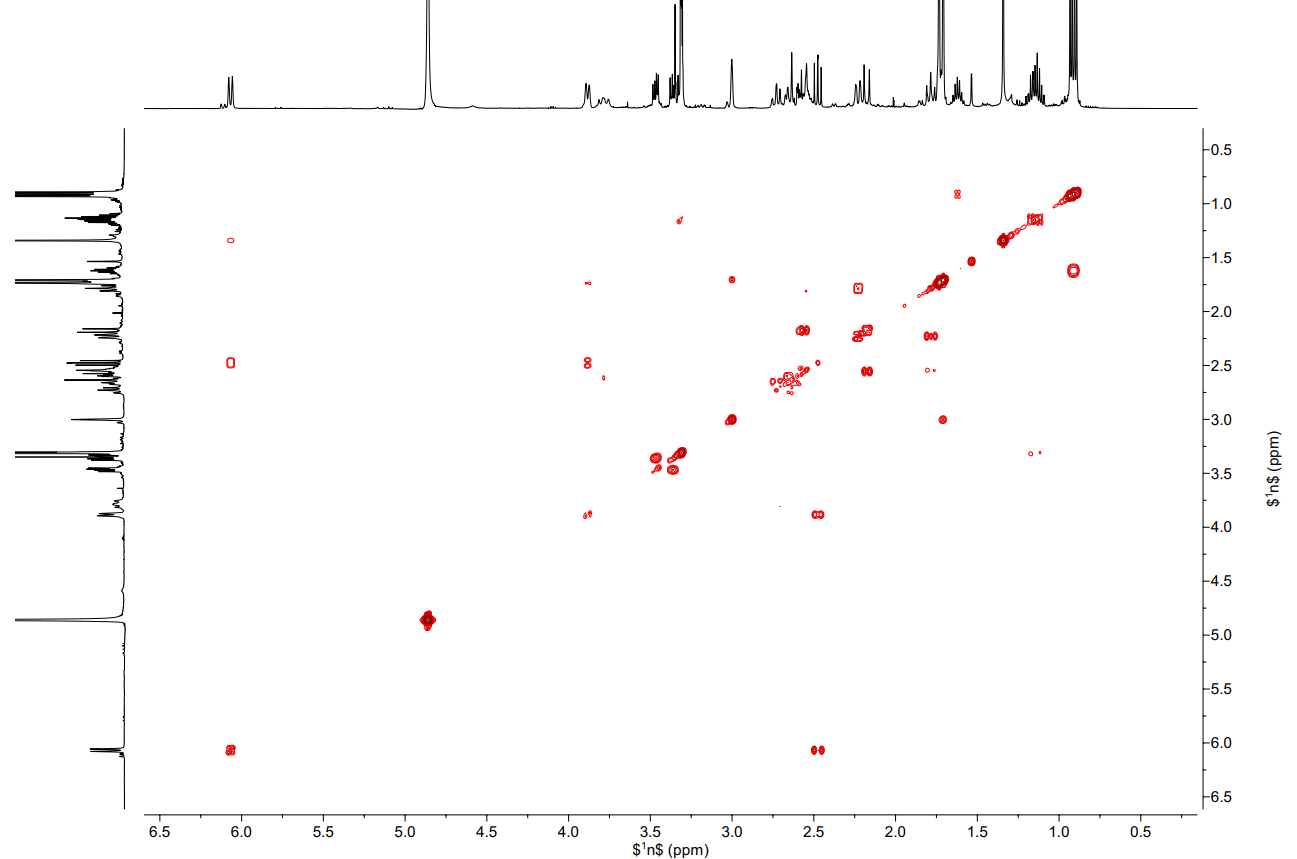

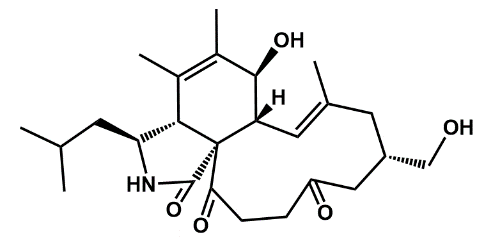


# Fig. S22. COSY spectrum of compound 3.


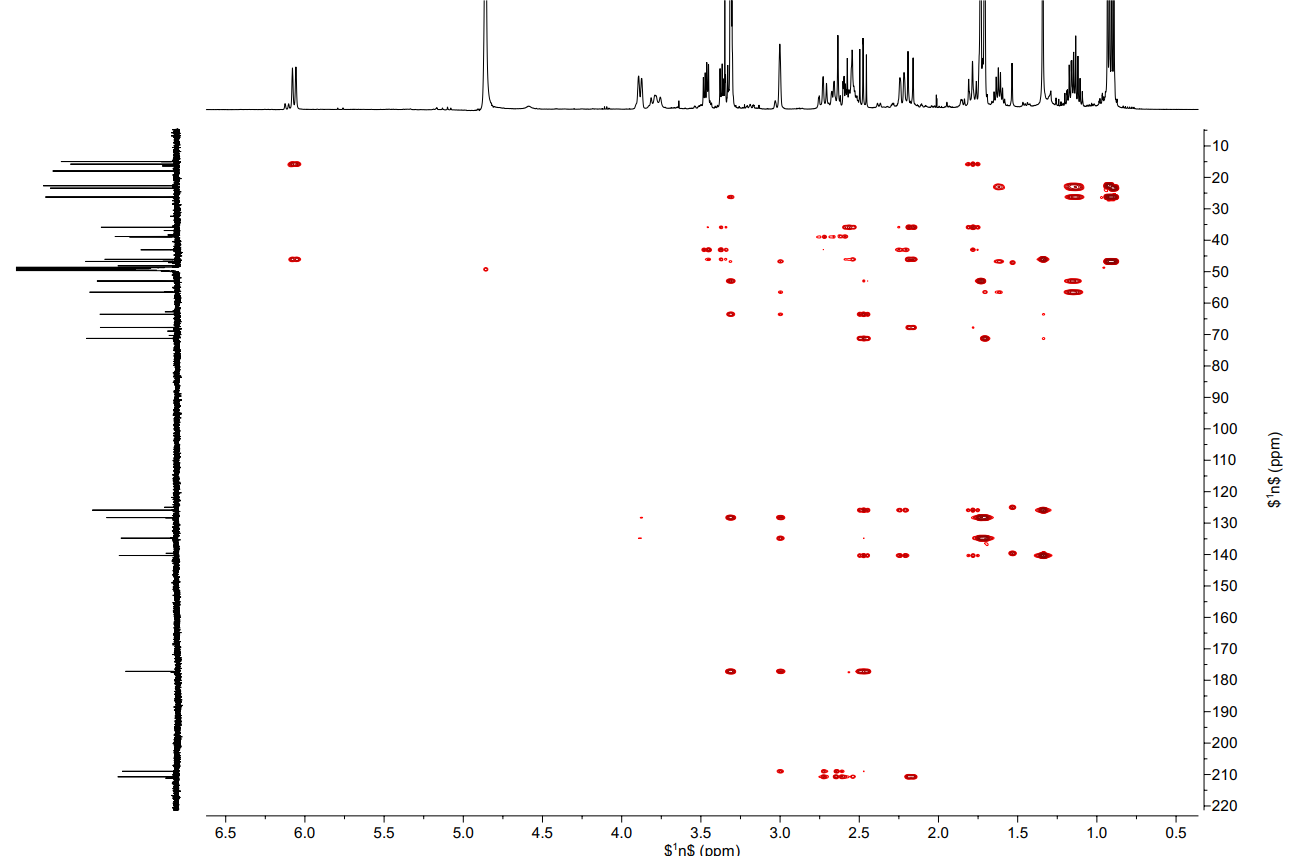

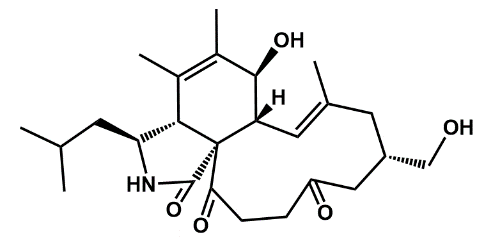


# Fig. S23. HMBC spectrum of compound 3.


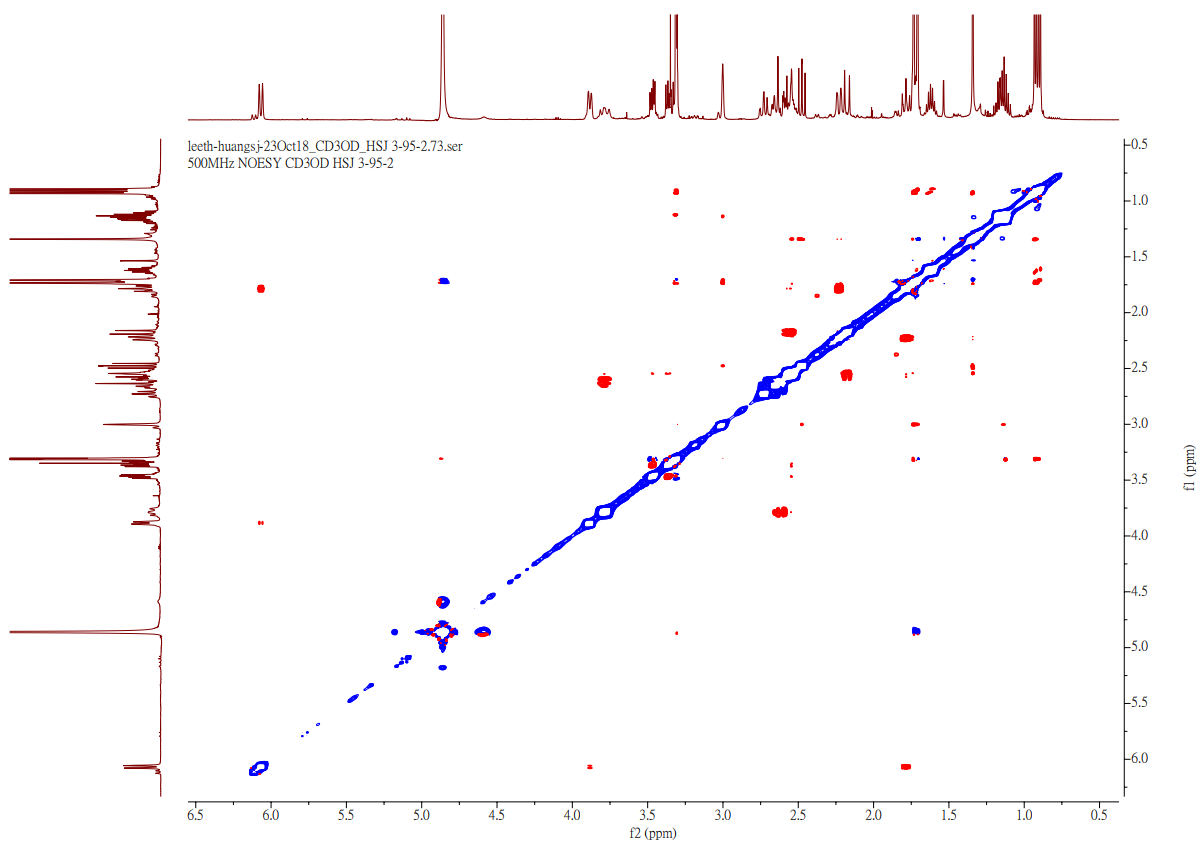

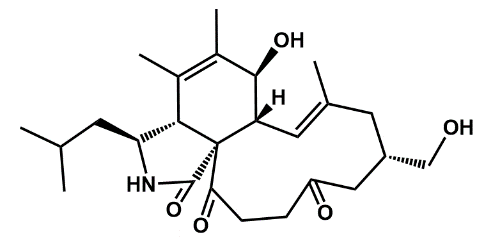


# Fig. S24. NOESY spectrum of compound 3.

# Table S1. Crystal data and experimental details for compound 1.

Crystal data

Empirical formula C25 H42 N O6.50

Formula weight 460.59

Crystal system Monoclinic

Space group P2**_1_**

Unit cell dimensions a = 9.5486(3) Å α= 90°.

b = 41.4842(15) Å β= 111.0752(19)°.

c = 13.2919(4) Å γ = 90°.

Volume 4912.9(3) Å3

Z 8

F(000) 2008

Density (calculated) 1.245 Mg/m3

Wavelength 1.54178 Å

Cell parameters reflections used 9563

Theta range for Cell parameters 3.20 to 74.82°.

Absorption coefficient 0.720 mm-1

Temperature 100(2) K

Crystal size 0.220 x 0.180 x 0.020 mm3

Data collection

Diffractometer Bruker AXS D8 VENTURE, PhotonIII_C28

Absorption correction Semi-empirical from equivalents

Max. and min. transmission 1.0000 and 0.8118

No. of measured reflections 107015

No. of independent reflections 19508 [R(int) = 0.0675]

No. of observed [I>2_igma(I)] 18170

Completeness to theta = 67.679° 99.1 %

Theta range for data collection 1.065 to 75.048°.

Refinement

Final R indices [I>2sigma(I)] R1 = 0.0557, wR2 = 0.1395

R indices (all data) R1 = 0.0620, wR2 = 0.1462

Goodness-of-fit on F2 0.974

No. of reflections 19508

No. of parameters 1199

No. of restraints 1

Absolute structure parameter 0.08(12)

Largest diff. peak and hole 0.395 and -0.303 e.Å-3

**Table S2.** Bond lengths [Å] and angles [°] for compound **1**.

_____________________________________________________

O(1)-C(21) 1.435(7)

O(1)-C(18) 1.436(7)

O(2)-C(21) 1.394(8)

O(3)-C(1) 1.239(8)

O(4)-C(14) 1.445(6)

O(5)-C(26) 1.427(8)

N(2)-C(1) 1.335(9)

N(2)-C(3) 1.468(8)

C(1)-C(9) 1.529(8)

C(3)-C(10) 1.513(9)

C(3)-C(4) 1.556(9)

C(4)-C(5) 1.542(9)

C(4)-C(9) 1.554(8)

C(5)-C(6) 1.532(9)

C(5)-C(11) 1.541(9)

C(6)-C(7) 1.325(10)

C(6)-C(12) 1.503(9)

C(7)-C(8) 1.497(8)

C(8)-C(13) 1.518(9)

C(8)-C(9) 1.572(9)

C(9)-C(21) 1.552(8)

C(10)-C(22) 1.526(10)

C(13)-C(18) 1.560(8)

C(13)-C(14) 1.567(9)

C(14)-C(15) 1.522(9)

C(14)-C(25) 1.531(9)

C(15)-C(16) 1.530(9)

C(16)-C(17) 1.552(8)

C(17)-C(18) 1.524(9)

C(18)-C(19) 1.552(8)

C(19)-C(20) 1.548(9)

C(20)-C(21) 1.544(9)

C(22)-C(24) 1.529(11)

C(22)-C(23) 1.530(11)

C(21)-O(1)-C(18) 105.9(5)

C(1)-N(2)-C(3) 115.7(5)

O(3)-C(1)-N(2) 125.0(6)

O(3)-C(1)-C(9) 125.2(5)

N(2)-C(1)-C(9) 109.8(5)

N(2)-C(3)-C(10) 112.7(6)

N(2)-C(3)-C(4) 103.5(5)

C(10)-C(3)-C(4) 113.7(5)

C(5)-C(4)-C(9) 112.4(5)

C(5)-C(4)-C(3) 111.7(5)

C(9)-C(4)-C(3) 106.7(5)

C(6)-C(5)-C(11) 115.8(6)

C(6)-C(5)-C(4) 108.4(5)

C(11)-C(5)-C(4) 111.6(6)

C(7)-C(6)-C(12) 123.7(6)

C(7)-C(6)-C(5) 115.7(6)

C(12)-C(6)-C(5) 120.7(6)

C(6)-C(7)-C(8) 118.5(6)

C(7)-C(8)-C(13) 120.6(5)

C(7)-C(8)-C(9) 106.9(5)

C(13)-C(8)-C(9) 111.5(5)

C(1)-C(9)-C(21) 108.1(5)

C(1)-C(9)-C(4) 104.0(5)

C(21)-C(9)-C(4) 113.3(5)

C(1)-C(9)-C(8) 111.4(5)

C(21)-C(9)-C(8) 109.1(5)

C(4)-C(9)-C(8) 110.9(5)

C(3)-C(10)-C(22) 116.3(6)

C(8)-C(13)-C(18) 108.4(5)

C(8)-C(13)-C(14) 116.0(5)

C(18)-C(13)-C(14) 112.9(5)

O(4)-C(14)-C(15) 107.9(5)

O(4)-C(14)-C(25) 104.3(5)

C(15)-C(14)-C(25) 110.7(5)

O(4)-C(14)-C(13) 108.1(5)

C(15)-C(14)-C(13) 109.3(5)

C(25)-C(14)-C(13) 116.1(5)

C(14)-C(15)-C(16) 113.6(5)

C(26)-C(16)-C(15) 111.4(5)

C(26)-C(16)-C(17) 110.2(5)

C(15)-C(16)-C(17) 109.5(5)

C(18)-C(17)-C(16) 113.6(5)

O(1)-C(18)-C(17) 107.0(5)

O(1)-C(18)-C(19) 101.3(4)

C(17)-C(18)-C(19) 113.5(5)

O(1)-C(18)-C(13) 108.7(5)

C(17)-C(18)-C(13) 110.9(5)

C(19)-C(18)-C(13) 114.6(5)

C(20)-C(19)-C(18) 104.0(5)

C(21)-C(20)-C(19) 103.7(5)

O(2)-C(21)-O(1) 110.2(5)

O(2)-C(21)-C(20) 115.6(5)

O(1)-C(21)-C(20) 102.5(5)

O(2)-C(21)-C(9) 107.7(5)

O(1)-C(21)-C(9) 107.3(4)

C(20)-C(21)-C(9) 113.2(5)

C(10)-C(22)-C(24) 114.3(6)

C(10)-C(22)-C(23) 109.8(7)

C(24)-C(22)-C(23) 111.2(7)

_________________________

# Table S3. CDOCKER energy and interaction pattern of potential inducible nitric oxide synthase (iNOS) inhibitors predicted by molecular docking analysis.

| Interactions | Interaction residues | Compounds | | | |
| --- | --- | --- | --- | --- | --- |
|  |  | **1** | **2** | **3** | *Curcumin |
| Cdocker interaction energy (mol /Kcal) |  | -44.0605 | -45.2659 | -46.4667 | -59.3552 |
| H-bond | ARG193 |  |  |  | **✓** |
|  | ARG260 |  |  |  | **✓** |
|  | ARG375 |  |  | **✓** |  |
|  | ARG382 |  |  |  | **✓** |
|  | ASP376 |  | **✓** | **✓** |  |
|  | GLN257 |  |  |  | **✓** |
|  | GLU371 | **✓** | **✓** | **✓** | **✓** |
|  | TYR341 |  |  |  | **✓** |
| Pi-Cation | ARG375 |  |  |  | **✓** |
| Pi Alkyl | ARG193 | **✓** | **✓** |  |  |
|  | CYS194 | **✓** | **✓** |  |  |
|  | PRO344 | **✓** | **✓** | **✓** |  |
|  | VAL346 | **✓** | **✓** |  |  |
|  |  |  |  |  |  |
| Alkyl | PHE363 | **✓** | **✓** |  |  |
|  | TRP188 | **✓** | **✓** |  |  |
|  | TRP367 |  |  | **✓** |  |
|  | TRP457 | **✓** | **✓** | **✓** | **✓** |

* Reference compound.
